# Dimethyl 2,2′-({2,2′-methyl­enebis[6-(2*H*-benzotriazol-2-yl)-4-(2,4,4-trimethyl­pentan-2-yl)-2,1-phenyl­ene]}di­oxy)diacetate

**DOI:** 10.1107/S1600536811006362

**Published:** 2011-02-26

**Authors:** Tahir Qadri, Itrat Anis, M. R. Shah, Seik Weng Ng

**Affiliations:** a H.E.J. Research Institute of Chemistry, International Center for Chemical and Biological Sciences, University of Karachi, Karachi 7527, Pakistan; b Department of Chemistry, University of Malaya, 50603 Kuala Lumpur, Malaysia

## Abstract

The asymmetric unit of the title compound, C_47_H_58_N_6_O_6_, comprises three independent mol­ecules, in one of which one *tert*-butyl group is disordered in a 1:1 ratio. The mol­ecule is a di(ar­yl)methane having two aliphatic and one *N*-heterocyclic substituent in each aryl ring. For the mol­ecule having the disordered *tert*-butyl group, the aryl rings make an angle of 115.3 (2)° at the methyl­ene carbon; one aryl ring is aligned at 42.0 (1)° with respect to the *N*-heterocyclic substituent and the other at 48.7 (1)° with respect to its substituent. The two ordered mol­ecules are disposed about a pseudo center of inversion. The pairs of twist angles in these two mol­ecules differ [52.7 (1) and 61.7 (1)°, and 29.1 (1) and 58.5 (1)°].

## Related literature

For a similar compound, see: Ali *et al.* (2011 ▶).
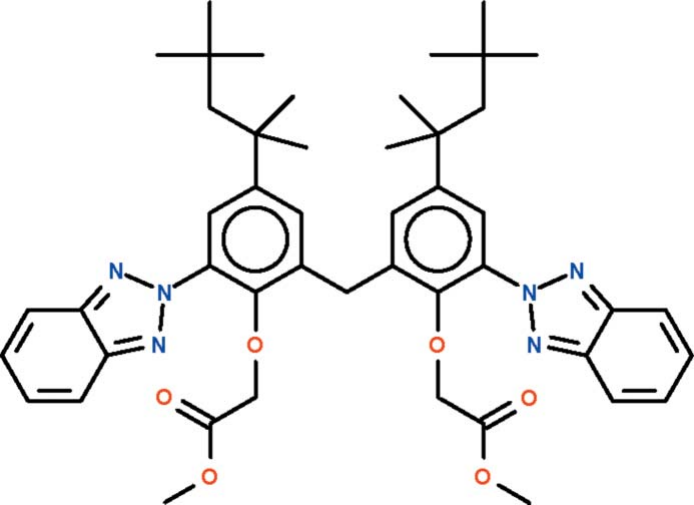


## Experimental

### 

#### Crystal data

C_47_H_58_N_6_O_6_
*M* *_r_* = 802.99
Triclinic, 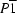

*a* = 19.4751 (6) Å
*b* = 19.7067 (6) Å
*c* = 20.6938 (7) Å
α = 113.881 (3)°
β = 113.983 (3)°
γ = 95.343 (2)°
*V* = 6311.7 (3) Å^3^
*Z* = 6
Mo *K*α radiation
μ = 0.09 mm^−1^
*T* = 100 K
0.30 × 0.20 × 0.10 mm

#### Data collection

Agilent SuperNova Dual diffractometer with an Atlas detector
Absorption correction: multi-scan (*CrysAlis PRO*; Agilent, 2010 ▶) *T*_min_ = 0.975, *T*_max_ = 0.992
55060 measured reflections
27973 independent reflections
17926 reflections with *I* > 2σ(*I*)
*R*_int_ = 0.044

#### Refinement

*R*[*F* ^2^ > 2σ(*F* ^2^)] = 0.070
*wR*(*F* ^2^) = 0.185
*S* = 1.07
27973 reflections
1642 parameters
43 restraints
H-atom parameters constrained
Δρ_max_ = 1.01 e Å^−3^
Δρ_min_ = −1.30 e Å^−3^

### 

Data collection: *CrysAlis PRO* (Agilent, 2010 ▶); cell refinement: *CrysAlis PRO*; data reduction: *CrysAlis PRO*; program(s) used to solve structure: *SHELXS97* (Sheldrick, 2008 ▶); program(s) used to refine structure: *SHELXL97* (Sheldrick, 2008 ▶); molecular graphics: *X-SEED* (Barbour, 2001 ▶); software used to prepare material for publication: *publCIF* (Westrip, 2010 ▶).

## Supplementary Material

Crystal structure: contains datablocks global, I. DOI: 10.1107/S1600536811006362/zs2091sup1.cif

Structure factors: contains datablocks I. DOI: 10.1107/S1600536811006362/zs2091Isup2.hkl

Additional supplementary materials:  crystallographic information; 3D view; checkCIF report

## Figures and Tables

**Fig. 1.**
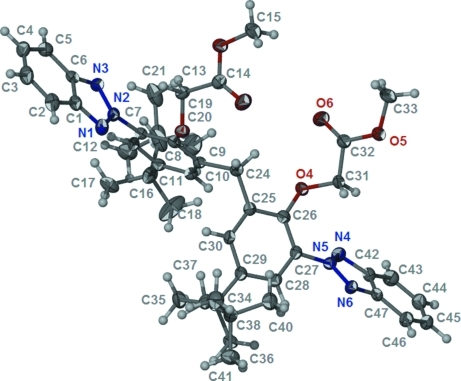
Thermal ellipsoid plot (Barbour, 2001) of one of the three independent molecules of C47H58N6O6 at the 70% probability level, with hydrogen atoms drawn as spheres of arbitrary radius. The disorder in the tert-butyl group is not shown.

**Fig. 2.**
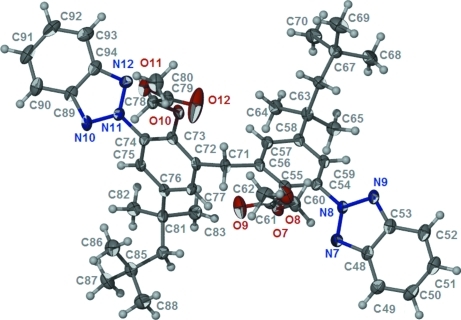
Thermal ellipsoid plot (Barbour, 2001) of the second independent C47H58N6O6 molecule. The molecule is ordered.

**Fig. 3.**
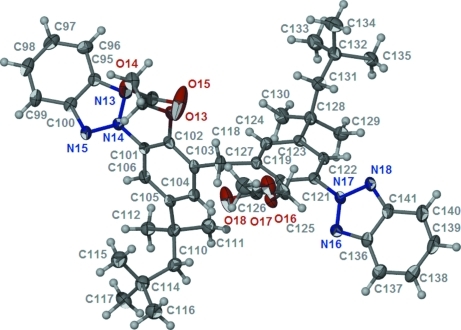
Thermal ellipsoid plot (Barbour, 2001) of the third independent C47H58N6O6 molecule. The molecule is ordered.

## supplementary crystallographic information

### Comment

Some background on di(aryl)methane compounds having oxyacetate substituents was presented in an earlier report (Ali *et al.*, 2011). The title compound also has an N-heterocyclic substituent in the rings (Scheme I). The asymmetric unit of C_47_H_58_N_6_O_6_ consists of three molecules (Figs. 1 to 3), one of which is disordered in one *tert*-butyl group in a 1:1 ratio. For the molecule having the disordered *tert*-butyl group, the aryl rings make an angle of 115.3 (2) ° at the methylene carbon with one aryl ring aligned at 42.0 (1) ° with respect to the N-heterocyclic substituent and the other at 48.7 (1) ° with respect to its substituent. The two ordered molecules are disposed about a false center-of-inversion with the pairs of twist angles in the other two molecules different [52.7 (1) and 61.7 (1)°; 29.1 (1) and 58.5 (1) °].

### Experimental

6,6'-Methylenebis(2-(2*H*-benzo[*d*][1,2,3]triazol-2-yl)-4-(2,4,4-trimethylpentan-2-yl)phenol) (0.10 g) and potassium carbonate (0.05 g) were dissolved in acetone (20 ml) at 323 K. Chloromethyl acetate (0.04 ml) was added and the reaction was stirred for 20 h. The progress of the reaction was monitored by thin layer chromatography (hexane:dichloromethane 60:40). The reaction was quenched by adding 1 M hydrochloric acid (10 ml). The aqueous phase was extracted with dichloromethane, the solvent evaporated and the crude product was recrystallized from dichloromethane (yield 80%).

### Refinement

Carbon-bound H-atoms were placed in calculated positions [C—H 0.95 to 0.98 Å, *U*_iso_(H) = 1.2 to 1.5*U*_eq_(C)] and were included in the refinement in the riding model approximation. One of the *tert*-butyl groups of one of the three independent molecules is disordered over two positions; the disorder could not be refined, and was assumed to be a 1:1 type of disorder. The *C*–C_methyl_ bond distance was restrained to 1.50±0.01 and the *C*···C_methyl_ distance to 2.35±0.01 Å. The displacement parameters of the primed atoms were set to those of the unprimed ones, and the anisotropic displacement parameters were restrained to be nearly isotropic. The anisotropic displacement parameters of the O12 atom were similarly restrained as the ellipsoid was too elongated. The final difference Fourier map had a large peak 0.34 Å from H21F and a hole 0.23 Å from C23'. One of the H-atoms of C23' (*i.e.*, H23E) is close to an ordered H18B atom at a distance of 1.78 Å; the interaction is probably an artifact of the disorder. The 'huge' version of *SHELXL97* was used for refining the structure.

### Crystal data

**Table:**

C_47_H_58_N_6_O_6_	*Z* = 6
*M**_r_* = 802.99	*F*(000) = 2580
Triclinic, *P*1	*D*_x_ = 1.268 Mg m^−^^3^
Hall symbol: -P 1	Mo *K*α radiation, λ = 0.71073 Å
*a* = 19.4751 (6) Å	Cell parameters from 12624 reflections
*b* = 19.7067 (6) Å	θ = 2.2–29.3°
*c* = 20.6938 (7) Å	µ = 0.09 mm^−^^1^
α = 113.881 (3)°	*T* = 100 K
β = 113.983 (3)°	Block, colorless
γ = 95.343 (2)°	0.30 × 0.20 × 0.10 mm
*V* = 6311.7 (3) Å^3^	

### Data collection

**Table:**

Agilent SuperNova Dual diffractometer with an Atlas detector	27973 independent reflections
Radiation source: SuperNova (Mo) X-ray Source	17926 reflections with *I* > 2σ(*I*)
Mirror	*R*_int_ = 0.044
Detector resolution: 10.4041 pixels mm^-1^	θ_max_ = 27.5°, θ_min_ = 2.2°
ω scans	*h* = −21→24
Absorption correction: multi-scan (*CrysAlis PRO*; Agilent, 2010)	*k* = −24→24
*T*_min_ = 0.975, *T*_max_ = 0.992	*l* = −26→26
55060 measured reflections	

### Refinement

**Table:**

Refinement on *F*^2^	Primary atom site location: structure-invariant direct methods
Least-squares matrix: full	Secondary atom site location: difference Fourier map
*R*[*F*^2^ > 2σ(*F*^2^)] = 0.070	Hydrogen site location: inferred from neighbouring sites
*wR*(*F*^2^) = 0.185	H-atom parameters constrained
*S* = 1.07	*w* = 1/[σ^2^(*F*_o_^2^) + (0.0603*P*)^2^ + 2.8599*P*] where *P* = (*F*_o_^2^ + 2*F*_c_^2^)/3
27973 reflections	(Δ/σ)_max_ = 0.001
1642 parameters	Δρ_max_ = 1.01 e Å^−^^3^
43 restraints	Δρ_min_ = −1.30 e Å^−^^3^

### Fractional atomic coordinates and isotropic or equivalent isotropic displacement parameters (Å^2^)

**Table:**

	*x*	*y*	*z*	*U*_iso_*/*U*_eq_	Occ. (<1)
O1	0.73152 (10)	0.56720 (9)	0.77982 (10)	0.0207 (4)	
O2	0.85523 (11)	0.45200 (10)	0.82251 (11)	0.0260 (4)	
O3	0.81080 (14)	0.47752 (13)	0.71920 (13)	0.0428 (6)	
O4	0.90742 (10)	0.73822 (10)	0.70957 (10)	0.0220 (4)	
O5	1.05002 (10)	0.64625 (11)	0.68355 (11)	0.0276 (4)	
O6	1.00785 (14)	0.66968 (15)	0.77371 (14)	0.0510 (6)	
O7	0.39861 (9)	0.90251 (9)	0.28506 (10)	0.0199 (4)	
O8	0.28531 (11)	1.03092 (10)	0.32003 (11)	0.0292 (4)	
O9	0.30210 (12)	0.97055 (12)	0.21200 (12)	0.0371 (5)	
O10	0.57413 (10)	1.07394 (9)	0.21479 (10)	0.0216 (4)	
O11	0.48334 (11)	1.21343 (10)	0.18105 (11)	0.0278 (4)	
O12	0.48700 (18)	1.16101 (16)	0.25872 (17)	0.0727 (9)	
O13	0.75124 (10)	0.58181 (10)	0.28035 (10)	0.0217 (4)	
O14	0.61810 (10)	0.68267 (10)	0.31868 (11)	0.0267 (4)	
O15	0.67145 (16)	0.67580 (18)	0.24041 (17)	0.0712 (9)	
O16	0.92310 (10)	0.75874 (9)	0.21179 (10)	0.0210 (4)	
O17	0.80837 (11)	0.88388 (11)	0.18169 (11)	0.0305 (4)	
O18	0.86067 (14)	0.86029 (13)	0.28557 (12)	0.0450 (6)	
N1	0.62974 (12)	0.57433 (12)	0.85424 (13)	0.0223 (5)	
N2	0.69904 (11)	0.62930 (11)	0.91052 (12)	0.0183 (4)	
N3	0.72977 (12)	0.63815 (12)	0.98517 (12)	0.0212 (5)	
N4	0.91394 (12)	0.84301 (12)	0.63749 (12)	0.0222 (5)	
N5	0.85063 (11)	0.78069 (11)	0.58118 (12)	0.0183 (4)	
N6	0.82883 (12)	0.75881 (12)	0.50399 (12)	0.0204 (4)	
N7	0.40599 (12)	0.80571 (12)	0.36251 (13)	0.0227 (5)	
N8	0.46353 (11)	0.87336 (11)	0.41788 (12)	0.0183 (4)	
N9	0.47710 (12)	0.90290 (12)	0.49412 (12)	0.0211 (5)	
N10	0.59904 (12)	1.00301 (12)	0.01474 (12)	0.0217 (5)	
N11	0.62368 (12)	1.02063 (11)	0.09157 (12)	0.0193 (4)	
N12	0.68861 (12)	1.08076 (11)	0.14875 (13)	0.0233 (5)	
N13	0.74878 (12)	0.46790 (12)	0.34612 (12)	0.0211 (5)	
N14	0.80490 (11)	0.53557 (11)	0.40402 (12)	0.0180 (4)	
N15	0.81346 (12)	0.56327 (12)	0.47805 (12)	0.0204 (4)	
N16	1.01935 (12)	0.75529 (12)	0.13324 (13)	0.0230 (5)	
N17	0.95471 (12)	0.69412 (11)	0.07873 (12)	0.0184 (4)	
N18	0.92752 (12)	0.67388 (12)	0.00071 (12)	0.0226 (5)	
C1	0.61365 (15)	0.54357 (14)	0.89600 (16)	0.0216 (5)	
C2	0.54644 (16)	0.48354 (15)	0.86945 (18)	0.0274 (6)	
H2	0.5051	0.4564	0.8148	0.033*	
C3	0.54465 (16)	0.46709 (15)	0.92705 (18)	0.0300 (6)	
H3	0.5008	0.4272	0.9118	0.036*	
C4	0.60611 (16)	0.50762 (16)	1.00875 (18)	0.0294 (6)	
H4	0.6017	0.4943	1.0465	0.035*	
C5	0.67123 (16)	0.56490 (16)	1.03508 (18)	0.0276 (6)	
H5	0.7121	0.5913	1.0899	0.033*	
C6	0.67516 (14)	0.58322 (14)	0.97698 (15)	0.0204 (5)	
C7	0.73651 (14)	0.67915 (14)	0.89162 (15)	0.0178 (5)	
C8	0.75003 (14)	0.64661 (13)	0.82607 (15)	0.0181 (5)	
C9	0.78143 (14)	0.69602 (14)	0.80547 (14)	0.0185 (5)	
C10	0.79896 (14)	0.77637 (14)	0.85179 (15)	0.0204 (5)	
H10	0.8196	0.8098	0.8371	0.025*	
C11	0.78746 (14)	0.80968 (14)	0.91889 (15)	0.0202 (5)	
C12	0.75618 (14)	0.75877 (14)	0.93844 (15)	0.0197 (5)	
H12	0.7484	0.7792	0.9843	0.024*	
C13	0.77144 (15)	0.52843 (14)	0.82148 (15)	0.0206 (5)	
H13A	0.7328	0.4917	0.8211	0.025*	
H13B	0.8097	0.5671	0.8785	0.025*	
C14	0.81423 (15)	0.48462 (14)	0.78077 (15)	0.0217 (5)	
C15	0.89703 (17)	0.40463 (16)	0.78880 (18)	0.0319 (6)	
H15A	0.9193	0.3782	0.8199	0.048*	
H15B	0.9398	0.4380	0.7914	0.048*	
H15C	0.8603	0.3657	0.7322	0.048*	
C16	0.81088 (16)	0.89799 (15)	0.97262 (16)	0.0260 (6)	
C17	0.74943 (16)	0.91726 (16)	1.00037 (17)	0.0307 (6)	
H17A	0.6963	0.8921	0.9532	0.046*	
H17B	0.7596	0.9739	1.0273	0.046*	
H17C	0.7533	0.8981	1.0382	0.046*	
C18	0.8124 (3)	0.93979 (18)	0.9253 (2)	0.0612 (11)	
H18A	0.8562	0.9347	0.9136	0.092*	
H18B	0.8195	0.9951	0.9574	0.092*	
H18C	0.7624	0.9165	0.8743	0.092*	
C19	0.89453 (14)	0.92226 (13)	1.04831 (15)	0.0367 (7)	
H19A	0.8865	0.8910	1.0734	0.044*	
H19B	0.9283	0.9013	1.0251	0.044*	
C20	0.94440 (15)	1.00016 (14)	1.11614 (16)	0.0381 (7)	
C21	0.9996 (3)	0.9840 (3)	1.1841 (2)	0.0383 (11)	0.50
H21A	1.0400	1.0329	1.2300	0.057*	0.50
H21B	1.0252	0.9469	1.1630	0.057*	0.50
H21C	0.9684	0.9621	1.2020	0.057*	0.50
C22	0.9083 (3)	1.0520 (3)	1.1501 (3)	0.0340 (13)	0.50
H22A	0.9490	1.0967	1.2015	0.051*	0.50
H22B	0.8718	1.0250	1.1598	0.051*	0.50
H22C	0.8792	1.0703	1.1127	0.051*	0.50
C23	1.0004 (3)	1.0347 (3)	1.0991 (3)	0.0436 (11)	0.50
H23A	1.0412	1.0808	1.1489	0.065*	0.50
H23B	0.9724	1.0500	1.0584	0.065*	0.50
H23C	1.0253	0.9964	1.0784	0.065*	0.50
C21'	1.0229 (2)	1.0038 (3)	1.1594 (3)	0.0383 (11)	0.50
H21D	1.0555	1.0585	1.1964	0.057*	0.50
H21E	1.0422	0.9790	1.1215	0.057*	0.50
H21F	1.0259	0.9766	1.1905	0.057*	0.50
C22'	0.9078 (3)	1.0294 (3)	1.1683 (3)	0.0340 (13)	0.50
H22D	0.9442	1.0788	1.2169	0.051*	0.50
H22E	0.8963	0.9910	1.1842	0.051*	0.50
H22F	0.8585	1.0378	1.1383	0.051*	0.50
C23'	0.9438 (3)	1.0593 (3)	1.0860 (3)	0.0436 (11)	0.50
H23D	0.9754	1.1115	1.1323	0.065*	0.50
H23E	0.8894	1.0586	1.0568	0.065*	0.50
H23F	0.9664	1.0456	1.0496	0.065*	0.50
C24	0.80119 (15)	0.66235 (15)	0.73768 (15)	0.0232 (6)	
H24A	0.7768	0.6050	0.7074	0.028*	
H24B	0.8592	0.6731	0.7619	0.028*	
C25	0.77457 (14)	0.69317 (13)	0.67822 (14)	0.0183 (5)	
C26	0.82864 (14)	0.72557 (14)	0.66195 (15)	0.0188 (5)	
C27	0.79985 (14)	0.74635 (13)	0.60139 (14)	0.0178 (5)	
C28	0.72023 (14)	0.73713 (14)	0.55893 (15)	0.0196 (5)	
H28	0.7025	0.7510	0.5173	0.024*	
C29	0.66579 (14)	0.70813 (14)	0.57598 (15)	0.0198 (5)	
C30	0.69503 (14)	0.68525 (14)	0.63524 (15)	0.0192 (5)	
H30	0.6588	0.6632	0.6467	0.023*	
C31	0.95160 (14)	0.70682 (15)	0.67052 (15)	0.0219 (5)	
H31A	0.9835	0.7484	0.6698	0.026*	
H31B	0.9153	0.6657	0.6139	0.026*	
C32	1.00501 (15)	0.67286 (15)	0.71661 (15)	0.0223 (5)	
C33	1.10627 (16)	0.61398 (16)	0.72295 (17)	0.0292 (6)	
H33A	1.1392	0.6007	0.6972	0.044*	
H33B	1.0779	0.5669	0.7181	0.044*	
H33C	1.1399	0.6525	0.7803	0.044*	
C34	0.57944 (14)	0.70691 (15)	0.53766 (15)	0.0215 (5)	
C35	0.56255 (16)	0.75144 (17)	0.60751 (17)	0.0317 (6)	
H35A	0.6016	0.8035	0.6443	0.048*	
H35B	0.5659	0.7227	0.6372	0.048*	
H35C	0.5094	0.7565	0.5854	0.048*	
C36	0.56638 (15)	0.75121 (15)	0.49003 (16)	0.0239 (6)	
H36A	0.6004	0.8055	0.5264	0.036*	
H36B	0.5110	0.7499	0.4665	0.036*	
H36C	0.5794	0.7267	0.4466	0.036*	
C37	0.52027 (15)	0.62313 (15)	0.48710 (15)	0.0245 (6)	
H37A	0.5399	0.5963	0.5191	0.029*	
H37B	0.4697	0.6289	0.4855	0.029*	
C38	0.49961 (15)	0.56603 (14)	0.39984 (16)	0.0221 (5)	
C39	0.45560 (16)	0.48536 (16)	0.37917 (18)	0.0327 (7)	
H39A	0.4910	0.4680	0.4144	0.049*	
H39B	0.4380	0.4482	0.3225	0.049*	
H39C	0.4096	0.4882	0.3874	0.049*	
C40	0.57346 (16)	0.55975 (16)	0.39092 (17)	0.0297 (6)	
H40A	0.6006	0.6095	0.3995	0.045*	
H40B	0.5580	0.5184	0.3364	0.045*	
H40C	0.6090	0.5473	0.4311	0.045*	
C41	0.44393 (16)	0.58696 (16)	0.33847 (16)	0.0296 (6)	
H41A	0.4725	0.6350	0.3450	0.044*	
H41B	0.3991	0.5949	0.3477	0.044*	
H41C	0.4246	0.5444	0.2835	0.044*	
C42	0.93656 (14)	0.86362 (14)	0.59234 (16)	0.0215 (5)	
C43	0.99901 (16)	0.92642 (15)	0.61611 (17)	0.0272 (6)	
H43	1.0353	0.9616	0.6716	0.033*	
C44	1.00488 (16)	0.93427 (16)	0.55597 (17)	0.0278 (6)	
H44	1.0463	0.9759	0.5700	0.033*	
C45	0.95081 (15)	0.88207 (15)	0.47293 (17)	0.0261 (6)	
H45	0.9570	0.8903	0.4332	0.031*	
C46	0.89053 (16)	0.82077 (15)	0.44817 (16)	0.0247 (6)	
H46	0.8547	0.7860	0.3925	0.030*	
C47	0.88406 (14)	0.81159 (14)	0.51006 (15)	0.0191 (5)	
C48	0.37877 (14)	0.78967 (15)	0.40707 (15)	0.0211 (5)	
C49	0.31958 (15)	0.72354 (16)	0.38296 (17)	0.0256 (6)	
H49	0.2899	0.6832	0.3282	0.031*	
C50	0.30716 (15)	0.72051 (16)	0.44238 (17)	0.0277 (6)	
H50	0.2682	0.6769	0.4287	0.033*	
C51	0.35113 (16)	0.78092 (16)	0.52372 (18)	0.0284 (6)	
H51	0.3405	0.7764	0.5630	0.034*	
C52	0.40800 (16)	0.84524 (16)	0.54826 (17)	0.0270 (6)	
H52	0.4368	0.8853	0.6032	0.032*	
C53	0.42230 (15)	0.84974 (14)	0.48800 (16)	0.0213 (5)	
C54	0.51288 (14)	0.90783 (13)	0.39646 (15)	0.0174 (5)	
C55	0.47908 (14)	0.92018 (13)	0.33010 (14)	0.0176 (5)	
C56	0.52797 (14)	0.94924 (13)	0.30702 (14)	0.0177 (5)	
C57	0.60935 (14)	0.96519 (13)	0.35216 (15)	0.0190 (5)	
H57	0.6427	0.9839	0.3359	0.023*	
C58	0.64420 (14)	0.95503 (13)	0.41988 (14)	0.0177 (5)	
C59	0.59371 (14)	0.92622 (13)	0.44160 (14)	0.0181 (5)	
H59	0.6152	0.9192	0.4879	0.022*	
C60	0.36237 (15)	0.94849 (15)	0.32643 (15)	0.0227 (6)	
H60A	0.4035	0.9906	0.3807	0.027*	
H60B	0.3276	0.9155	0.3336	0.027*	
C61	0.31459 (14)	0.98341 (14)	0.27784 (15)	0.0218 (5)	
C62	0.23258 (16)	1.06462 (16)	0.27908 (18)	0.0327 (7)	
H62A	0.2064	1.0894	0.3098	0.049*	
H62B	0.1927	1.0235	0.2247	0.049*	
H62C	0.2629	1.1038	0.2750	0.049*	
C63	0.73427 (14)	0.97618 (14)	0.47100 (15)	0.0200 (5)	
C64	0.77201 (15)	0.96583 (15)	0.41638 (16)	0.0246 (6)	
H64A	0.7442	0.9143	0.3680	0.037*	
H64B	0.8277	0.9703	0.4461	0.037*	
H64C	0.7681	1.0064	0.4003	0.037*	
C65	0.75389 (15)	0.92045 (14)	0.50543 (16)	0.0241 (6)	
H65A	0.7271	0.8664	0.4614	0.036*	
H65B	0.7360	0.9297	0.5453	0.036*	
H65C	0.8111	0.9297	0.5316	0.036*	
C66	0.76084 (14)	1.06264 (14)	0.53770 (15)	0.0214 (5)	
H66A	0.7497	1.0936	0.5092	0.026*	
H66B	0.7245	1.0655	0.5603	0.026*	
C67	0.84515 (15)	1.10735 (15)	0.61117 (16)	0.0236 (6)	
C68	0.86502 (17)	1.07819 (17)	0.67249 (17)	0.0359 (7)	
H68A	0.9133	1.1154	0.7226	0.054*	
H68B	0.8731	1.0272	0.6502	0.054*	
H68C	0.8214	1.0732	0.6841	0.054*	
C69	0.84695 (17)	1.19215 (16)	0.65328 (18)	0.0368 (7)	
H69A	0.8339	1.2123	0.6150	0.055*	
H69B	0.8999	1.2237	0.6994	0.055*	
H69C	0.8083	1.1949	0.6724	0.055*	
C70	0.91048 (16)	1.10661 (17)	0.58821 (18)	0.0341 (7)	
H70A	0.8980	1.1234	0.5473	0.051*	
H70B	0.9144	1.0537	0.5663	0.051*	
H70C	0.9609	1.1423	0.6364	0.051*	
C71	0.49259 (15)	0.96613 (14)	0.23754 (14)	0.0198 (5)	
H71A	0.4999	1.0228	0.2599	0.024*	
H71B	0.4350	0.9388	0.2057	0.024*	
C72	0.52706 (14)	0.94218 (14)	0.18089 (14)	0.0174 (5)	
C73	0.56231 (14)	0.99634 (13)	0.16717 (14)	0.0180 (5)	
C74	0.58637 (14)	0.96950 (13)	0.10944 (14)	0.0176 (5)	
C75	0.57821 (14)	0.89075 (13)	0.06679 (14)	0.0182 (5)	
H75	0.5948	0.8741	0.0272	0.022*	
C76	0.54607 (14)	0.83626 (14)	0.08144 (14)	0.0186 (5)	
C77	0.52043 (14)	0.86404 (14)	0.13834 (14)	0.0186 (5)	
H77	0.4972	0.8277	0.1486	0.022*	
C78	0.54868 (15)	1.11972 (14)	0.17648 (15)	0.0208 (5)	
H78A	0.5147	1.0854	0.1174	0.025*	
H78B	0.5950	1.1556	0.1858	0.025*	
C79	0.50314 (16)	1.16571 (15)	0.21116 (16)	0.0250 (6)	
C80	0.43948 (16)	1.26189 (16)	0.20948 (17)	0.0290 (6)	
H80A	0.4242	1.2910	0.1799	0.044*	
H80B	0.4726	1.2986	0.2675	0.044*	
H80C	0.3920	1.2289	0.2003	0.044*	
C81	0.54547 (15)	0.75078 (14)	0.04287 (15)	0.0200 (5)	
C82	0.58856 (15)	0.73806 (14)	−0.00630 (16)	0.0231 (5)	
H82A	0.5636	0.7526	−0.0484	0.035*	
H82B	0.5858	0.6830	−0.0316	0.035*	
H82C	0.6440	0.7704	0.0295	0.035*	
C83	0.59283 (16)	0.73531 (15)	0.11333 (16)	0.0268 (6)	
H83A	0.6455	0.7739	0.1487	0.040*	
H83B	0.5979	0.6828	0.0915	0.040*	
H83C	0.5651	0.7394	0.1444	0.040*	
C84	0.46125 (15)	0.69253 (14)	−0.00552 (15)	0.0228 (5)	
H84A	0.4676	0.6423	−0.0084	0.027*	
H84B	0.4355	0.7113	0.0280	0.027*	
C85	0.40164 (15)	0.67374 (15)	−0.09188 (16)	0.0234 (6)	
C86	0.39489 (16)	0.74684 (16)	−0.09990 (17)	0.0300 (6)	
H86A	0.4455	0.7747	−0.0901	0.045*	
H86B	0.3812	0.7808	−0.0602	0.045*	
H86C	0.3536	0.7319	−0.1546	0.045*	
C87	0.42033 (16)	0.61839 (16)	−0.15518 (16)	0.0289 (6)	
H87A	0.4678	0.6464	−0.1507	0.043*	
H87B	0.3757	0.5991	−0.2095	0.043*	
H87C	0.4295	0.5743	−0.1459	0.043*	
C88	0.32046 (16)	0.63051 (16)	−0.11078 (18)	0.0323 (7)	
H88A	0.3041	0.6655	−0.0743	0.049*	
H88B	0.3238	0.5848	−0.1031	0.049*	
H88C	0.2817	0.6137	−0.1670	0.049*	
C89	0.65312 (15)	1.05808 (14)	0.02252 (16)	0.0222 (5)	
C90	0.66150 (16)	1.06765 (17)	−0.03827 (18)	0.0292 (6)	
H90	0.6250	1.0344	−0.0941	0.035*	
C91	0.72441 (17)	1.12683 (17)	−0.01283 (19)	0.0330 (7)	
H91	0.7321	1.1346	−0.0521	0.040*	
C92	0.77882 (17)	1.17726 (16)	0.07037 (19)	0.0324 (7)	
H92	0.8209	1.2187	0.0850	0.039*	
C93	0.77294 (16)	1.16841 (15)	0.13056 (18)	0.0289 (6)	
H93	0.8101	1.2019	0.1862	0.035*	
C94	0.70838 (15)	1.10661 (14)	0.10492 (16)	0.0230 (6)	
C95	0.71648 (14)	0.44991 (14)	0.38654 (15)	0.0202 (5)	
C96	0.65517 (15)	0.38359 (15)	0.35888 (17)	0.0262 (6)	
H96	0.6273	0.3438	0.3038	0.031*	
C97	0.63829 (16)	0.37978 (16)	0.41544 (18)	0.0292 (6)	
H97	0.5974	0.3363	0.3991	0.035*	
C98	0.67970 (16)	0.43859 (16)	0.49754 (18)	0.0300 (6)	
H98	0.6662	0.4329	0.5347	0.036*	
C99	0.73827 (16)	0.50319 (15)	0.52530 (17)	0.0270 (6)	
H99	0.7653	0.5425	0.5806	0.032*	
C100	0.75691 (15)	0.50890 (14)	0.46785 (16)	0.0213 (5)	
C101	0.85790 (14)	0.57377 (13)	0.38853 (14)	0.0173 (5)	
C102	0.83023 (14)	0.59678 (13)	0.32965 (14)	0.0179 (5)	
C103	0.88495 (14)	0.63311 (13)	0.31684 (14)	0.0180 (5)	
C104	0.96456 (14)	0.64260 (14)	0.36162 (15)	0.0196 (5)	
H10B	1.0013	0.6667	0.3521	0.024*	
C105	0.99330 (14)	0.61855 (14)	0.41971 (15)	0.0198 (5)	
C106	0.93774 (14)	0.58513 (14)	0.43318 (15)	0.0202 (5)	
H10C	0.9549	0.5699	0.4737	0.024*	
C107	0.70480 (14)	0.60898 (14)	0.31805 (15)	0.0205 (5)	
H10D	0.7390	0.6396	0.3775	0.025*	
H10E	0.6648	0.5641	0.3056	0.025*	
C108	0.66457 (15)	0.65916 (15)	0.28687 (16)	0.0240 (6)	
C109	0.57343 (16)	0.72991 (16)	0.29222 (17)	0.0283 (6)	
H10F	0.5436	0.7459	0.3212	0.042*	
H10G	0.5367	0.6995	0.2339	0.042*	
H10H	0.6098	0.7763	0.3037	0.042*	
C110	1.08055 (14)	0.62109 (15)	0.45923 (16)	0.0232 (6)	
C111	1.09692 (16)	0.57357 (16)	0.38952 (17)	0.0310 (6)	
H11A	1.1503	0.5693	0.4122	0.047*	
H11B	1.0925	0.6001	0.3575	0.047*	
H11C	1.0583	0.5213	0.3547	0.047*	
C112	1.09561 (15)	0.58104 (15)	0.51050 (16)	0.0250 (6)	
H11D	1.1517	0.5852	0.5361	0.038*	
H11E	1.0639	0.5259	0.4761	0.038*	
H11F	1.0809	0.6062	0.5525	0.038*	
C113	1.13838 (15)	0.70448 (15)	0.50574 (16)	0.0257 (6)	
H11G	1.1183	0.7287	0.4711	0.031*	
H11H	1.1894	0.6987	0.5087	0.031*	
C114	1.15775 (16)	0.76523 (15)	0.59237 (17)	0.0269 (6)	
C115	1.08274 (16)	0.76919 (17)	0.59879 (18)	0.0340 (7)	
H11I	1.0963	0.8116	0.6521	0.051*	
H11J	1.0573	0.7196	0.5918	0.051*	
H11K	1.0465	0.7790	0.5565	0.051*	
C116	1.19896 (17)	0.84487 (16)	0.60803 (18)	0.0326 (7)	
H11L	1.2170	0.8842	0.6641	0.049*	
H11M	1.1618	0.8595	0.5711	0.049*	
H11N	1.2444	0.8418	0.5989	0.049*	
C117	1.21601 (17)	0.74909 (16)	0.65698 (17)	0.0319 (6)	
H11O	1.2350	0.7936	0.7108	0.048*	
H11P	1.2609	0.7412	0.6478	0.048*	
H11Q	1.1894	0.7021	0.6536	0.048*	
C118	0.86001 (16)	0.66527 (15)	0.25868 (15)	0.0229 (6)	
H11R	0.8859	0.7224	0.2896	0.027*	
H11S	0.8022	0.6561	0.2348	0.027*	
C119	0.87942 (14)	0.63072 (14)	0.19051 (14)	0.0188 (5)	
C120	0.90770 (14)	0.67926 (13)	0.16729 (14)	0.0185 (5)	
C121	0.92115 (14)	0.64627 (14)	0.10172 (15)	0.0181 (5)	
C122	0.90568 (14)	0.56650 (13)	0.05851 (15)	0.0183 (5)	
H122	0.9137	0.5455	0.0129	0.022*	
C123	0.87852 (14)	0.51687 (14)	0.08116 (15)	0.0185 (5)	
C124	0.86603 (14)	0.55079 (14)	0.14746 (15)	0.0199 (5)	
H124	0.8476	0.5179	0.1640	0.024*	
C125	0.87848 (16)	0.79448 (14)	0.16965 (15)	0.0231 (6)	
H12D	0.8330	0.7541	0.1174	0.028*	
H12E	0.9118	0.8240	0.1582	0.028*	
C126	0.84944 (15)	0.84879 (14)	0.22054 (16)	0.0228 (5)	
C127	0.77839 (17)	0.93978 (16)	0.22430 (18)	0.0320 (7)	
H12F	0.7571	0.9680	0.1951	0.048*	
H12G	0.8213	0.9768	0.2790	0.048*	
H12H	0.7365	0.9124	0.2278	0.048*	
C128	0.85950 (15)	0.42822 (14)	0.03149 (15)	0.0224 (5)	
C129	0.91741 (15)	0.41108 (14)	−0.00175 (16)	0.0251 (6)	
H12I	0.9718	0.4395	0.0427	0.038*	
H12J	0.9070	0.4279	−0.0425	0.038*	
H12K	0.9104	0.3550	−0.0266	0.038*	
C130	0.87054 (17)	0.39182 (15)	0.08632 (16)	0.0288 (6)	
H13C	0.9227	0.4198	0.1348	0.043*	
H13D	0.8666	0.3369	0.0571	0.043*	
H13E	0.8295	0.3954	0.1023	0.043*	
C131	0.77218 (15)	0.40095 (14)	−0.03718 (16)	0.0246 (6)	
H13F	0.7399	0.4126	−0.0098	0.030*	
H13G	0.7700	0.4361	−0.0607	0.030*	
C132	0.72797 (15)	0.31810 (15)	−0.10902 (16)	0.0238 (6)	
C133	0.72679 (18)	0.25482 (16)	−0.08463 (17)	0.0337 (7)	
H13H	0.7800	0.2500	−0.0620	0.051*	
H13I	0.6901	0.2050	−0.1323	0.051*	
H13J	0.7095	0.2688	−0.0439	0.051*	
C134	0.64202 (16)	0.31588 (17)	−0.15380 (18)	0.0361 (7)	
H13K	0.6110	0.2637	−0.2004	0.054*	
H13L	0.6403	0.3544	−0.1725	0.054*	
H13M	0.6199	0.3281	−0.1170	0.054*	
C135	0.75888 (17)	0.29649 (16)	−0.16929 (16)	0.0322 (7)	
H13N	0.8105	0.2890	−0.1454	0.048*	
H13O	0.7643	0.3385	−0.1824	0.048*	
H13P	0.7217	0.2481	−0.2188	0.048*	
C136	1.03623 (15)	0.77844 (14)	0.08674 (15)	0.0210 (5)	
C137	1.09976 (16)	0.83920 (15)	0.10892 (18)	0.0277 (6)	
H137	1.1384	0.8731	0.1639	0.033*	
C138	1.10304 (17)	0.84698 (16)	0.04738 (18)	0.0304 (6)	
H138	1.1445	0.8878	0.0600	0.036*	
C139	1.04599 (17)	0.79553 (18)	−0.03502 (18)	0.0339 (7)	
H139	1.0510	0.8028	−0.0758	0.041*	
C140	0.98468 (16)	0.73641 (18)	−0.05786 (18)	0.0324 (7)	
H140	0.9471	0.7023	−0.1132	0.039*	
C141	0.97961 (15)	0.72811 (15)	0.00499 (15)	0.0221 (5)	

### Atomic displacement parameters (Å^2^)

**Table:**

	*U*^11^	*U*^22^	*U*^33^	*U*^12^	*U*^13^	*U*^23^
O1	0.0247 (9)	0.0205 (9)	0.0153 (9)	0.0093 (7)	0.0087 (8)	0.0078 (8)
O2	0.0320 (10)	0.0294 (10)	0.0244 (10)	0.0183 (8)	0.0162 (9)	0.0155 (9)
O3	0.0683 (16)	0.0575 (14)	0.0406 (13)	0.0451 (12)	0.0422 (13)	0.0352 (12)
O4	0.0176 (9)	0.0315 (10)	0.0157 (9)	0.0107 (7)	0.0077 (8)	0.0103 (8)
O5	0.0283 (10)	0.0399 (11)	0.0280 (11)	0.0207 (9)	0.0182 (9)	0.0214 (9)
O6	0.0688 (16)	0.0883 (18)	0.0504 (15)	0.0602 (14)	0.0473 (14)	0.0545 (14)
O7	0.0174 (9)	0.0257 (9)	0.0160 (9)	0.0088 (7)	0.0087 (8)	0.0084 (8)
O8	0.0295 (10)	0.0328 (11)	0.0263 (11)	0.0179 (8)	0.0150 (9)	0.0123 (9)
O9	0.0473 (13)	0.0524 (13)	0.0265 (11)	0.0323 (11)	0.0212 (10)	0.0251 (10)
O10	0.0330 (10)	0.0176 (9)	0.0155 (9)	0.0111 (7)	0.0123 (8)	0.0080 (7)
O11	0.0395 (11)	0.0307 (10)	0.0317 (11)	0.0222 (9)	0.0251 (10)	0.0209 (9)
O12	0.137 (3)	0.096 (2)	0.094 (2)	0.0952 (19)	0.102 (2)	0.0811 (18)
O13	0.0184 (9)	0.0306 (10)	0.0155 (9)	0.0113 (7)	0.0083 (8)	0.0096 (8)
O14	0.0317 (10)	0.0347 (11)	0.0305 (11)	0.0217 (9)	0.0206 (9)	0.0225 (9)
O15	0.094 (2)	0.133 (2)	0.103 (2)	0.096 (2)	0.0862 (19)	0.107 (2)
O16	0.0269 (10)	0.0183 (9)	0.0150 (9)	0.0091 (7)	0.0080 (8)	0.0075 (7)
O17	0.0409 (12)	0.0316 (11)	0.0276 (11)	0.0228 (9)	0.0182 (10)	0.0178 (9)
O18	0.0670 (16)	0.0585 (14)	0.0292 (12)	0.0445 (12)	0.0306 (12)	0.0256 (11)
N1	0.0236 (11)	0.0204 (11)	0.0210 (12)	0.0052 (9)	0.0131 (10)	0.0067 (9)
N2	0.0188 (11)	0.0200 (11)	0.0161 (11)	0.0063 (8)	0.0097 (9)	0.0075 (9)
N3	0.0223 (11)	0.0255 (11)	0.0190 (12)	0.0098 (9)	0.0112 (10)	0.0120 (10)
N4	0.0182 (11)	0.0257 (12)	0.0173 (11)	0.0020 (9)	0.0063 (10)	0.0093 (10)
N5	0.0164 (10)	0.0215 (11)	0.0143 (11)	0.0045 (8)	0.0058 (9)	0.0084 (9)
N6	0.0246 (11)	0.0235 (11)	0.0176 (11)	0.0103 (9)	0.0128 (10)	0.0109 (9)
N7	0.0208 (11)	0.0267 (12)	0.0193 (12)	0.0043 (9)	0.0095 (10)	0.0112 (10)
N8	0.0188 (11)	0.0220 (11)	0.0163 (11)	0.0062 (8)	0.0091 (9)	0.0106 (9)
N9	0.0274 (12)	0.0228 (11)	0.0187 (11)	0.0098 (9)	0.0147 (10)	0.0111 (9)
N10	0.0247 (12)	0.0283 (12)	0.0183 (11)	0.0113 (9)	0.0121 (10)	0.0145 (10)
N11	0.0240 (11)	0.0187 (11)	0.0198 (11)	0.0072 (9)	0.0130 (10)	0.0109 (9)
N12	0.0285 (12)	0.0176 (11)	0.0233 (12)	0.0039 (9)	0.0148 (11)	0.0080 (10)
N13	0.0182 (11)	0.0227 (11)	0.0195 (11)	0.0036 (9)	0.0072 (10)	0.0103 (9)
N14	0.0200 (11)	0.0178 (10)	0.0158 (11)	0.0057 (8)	0.0085 (9)	0.0081 (9)
N15	0.0233 (11)	0.0227 (11)	0.0183 (11)	0.0091 (9)	0.0128 (10)	0.0097 (9)
N16	0.0258 (12)	0.0199 (11)	0.0192 (12)	0.0024 (9)	0.0111 (10)	0.0069 (9)
N17	0.0199 (11)	0.0204 (11)	0.0146 (11)	0.0060 (8)	0.0078 (9)	0.0089 (9)
N18	0.0225 (11)	0.0307 (12)	0.0167 (11)	0.0095 (9)	0.0102 (10)	0.0124 (10)
C1	0.0237 (13)	0.0196 (13)	0.0269 (15)	0.0100 (10)	0.0164 (12)	0.0110 (11)
C2	0.0268 (14)	0.0236 (14)	0.0327 (16)	0.0068 (11)	0.0180 (13)	0.0112 (12)
C3	0.0332 (16)	0.0244 (14)	0.0466 (19)	0.0115 (12)	0.0284 (15)	0.0199 (14)
C4	0.0368 (16)	0.0327 (15)	0.0424 (18)	0.0200 (13)	0.0291 (15)	0.0268 (14)
C5	0.0299 (15)	0.0319 (15)	0.0350 (17)	0.0161 (12)	0.0197 (14)	0.0229 (13)
C6	0.0204 (13)	0.0224 (13)	0.0242 (14)	0.0122 (10)	0.0123 (12)	0.0135 (11)
C7	0.0163 (12)	0.0200 (12)	0.0195 (13)	0.0076 (10)	0.0090 (11)	0.0110 (11)
C8	0.0172 (12)	0.0188 (12)	0.0167 (13)	0.0076 (10)	0.0075 (11)	0.0078 (10)
C9	0.0172 (12)	0.0249 (13)	0.0133 (12)	0.0085 (10)	0.0065 (11)	0.0097 (11)
C10	0.0205 (13)	0.0248 (13)	0.0211 (14)	0.0089 (10)	0.0109 (12)	0.0144 (11)
C11	0.0193 (13)	0.0222 (13)	0.0171 (13)	0.0083 (10)	0.0069 (11)	0.0095 (11)
C12	0.0214 (13)	0.0260 (13)	0.0157 (13)	0.0101 (10)	0.0117 (11)	0.0102 (11)
C13	0.0271 (14)	0.0194 (13)	0.0183 (13)	0.0093 (10)	0.0118 (12)	0.0105 (11)
C14	0.0231 (13)	0.0208 (13)	0.0172 (13)	0.0047 (10)	0.0088 (12)	0.0075 (11)
C15	0.0319 (16)	0.0355 (16)	0.0344 (17)	0.0195 (13)	0.0188 (14)	0.0177 (14)
C16	0.0336 (15)	0.0209 (13)	0.0229 (15)	0.0052 (11)	0.0159 (13)	0.0087 (12)
C17	0.0257 (15)	0.0268 (15)	0.0293 (16)	0.0136 (12)	0.0084 (13)	0.0088 (13)
C18	0.131 (4)	0.0254 (17)	0.053 (2)	0.028 (2)	0.061 (3)	0.0236 (17)
C19	0.0235 (15)	0.0345 (16)	0.0385 (18)	0.0084 (12)	0.0163 (15)	0.0052 (14)
C20	0.0406 (18)	0.0256 (15)	0.0368 (18)	−0.0011 (13)	0.0236 (16)	0.0030 (13)
C21	0.027 (2)	0.034 (2)	0.038 (3)	0.0084 (17)	0.0177 (19)	0.0016 (18)
C22	0.0313 (18)	0.035 (3)	0.020 (2)	0.0114 (19)	0.0057 (18)	0.0071 (16)
C23	0.040 (2)	0.045 (2)	0.038 (2)	0.0011 (18)	0.017 (2)	0.017 (2)
C21'	0.027 (2)	0.034 (2)	0.038 (3)	0.0084 (17)	0.0177 (19)	0.0016 (18)
C22'	0.0313 (18)	0.035 (3)	0.020 (2)	0.0114 (19)	0.0057 (18)	0.0071 (16)
C23'	0.040 (2)	0.045 (2)	0.038 (2)	0.0011 (18)	0.017 (2)	0.017 (2)
C24	0.0272 (14)	0.0305 (14)	0.0214 (14)	0.0163 (11)	0.0150 (12)	0.0161 (12)
C25	0.0232 (13)	0.0169 (12)	0.0160 (13)	0.0091 (10)	0.0114 (11)	0.0065 (10)
C26	0.0194 (13)	0.0191 (12)	0.0152 (13)	0.0075 (10)	0.0078 (11)	0.0063 (10)
C27	0.0203 (13)	0.0175 (12)	0.0166 (13)	0.0063 (10)	0.0108 (11)	0.0072 (10)
C28	0.0221 (13)	0.0207 (13)	0.0157 (13)	0.0066 (10)	0.0092 (11)	0.0085 (11)
C29	0.0205 (13)	0.0229 (13)	0.0167 (13)	0.0085 (10)	0.0107 (11)	0.0082 (11)
C30	0.0209 (13)	0.0218 (13)	0.0198 (13)	0.0078 (10)	0.0132 (11)	0.0108 (11)
C31	0.0194 (13)	0.0320 (14)	0.0186 (14)	0.0124 (11)	0.0114 (12)	0.0130 (12)
C32	0.0220 (13)	0.0261 (14)	0.0190 (14)	0.0097 (11)	0.0100 (12)	0.0107 (11)
C33	0.0291 (15)	0.0311 (15)	0.0329 (16)	0.0180 (12)	0.0154 (14)	0.0183 (13)
C34	0.0190 (13)	0.0293 (14)	0.0197 (14)	0.0091 (11)	0.0103 (12)	0.0137 (12)
C35	0.0261 (15)	0.0461 (18)	0.0264 (16)	0.0161 (13)	0.0162 (14)	0.0163 (14)
C36	0.0216 (13)	0.0278 (14)	0.0240 (14)	0.0117 (11)	0.0107 (12)	0.0136 (12)
C37	0.0197 (13)	0.0324 (15)	0.0240 (15)	0.0058 (11)	0.0113 (12)	0.0157 (12)
C38	0.0232 (13)	0.0237 (13)	0.0230 (14)	0.0087 (11)	0.0130 (12)	0.0126 (11)
C39	0.0289 (15)	0.0346 (16)	0.0366 (17)	0.0075 (12)	0.0160 (14)	0.0195 (14)
C40	0.0291 (15)	0.0312 (15)	0.0334 (17)	0.0100 (12)	0.0184 (14)	0.0164 (13)
C41	0.0313 (15)	0.0275 (15)	0.0224 (15)	0.0109 (12)	0.0085 (13)	0.0098 (12)
C42	0.0201 (13)	0.0247 (13)	0.0241 (14)	0.0091 (10)	0.0130 (12)	0.0129 (12)
C43	0.0246 (14)	0.0276 (14)	0.0256 (15)	0.0058 (11)	0.0105 (13)	0.0118 (12)
C44	0.0261 (14)	0.0298 (15)	0.0358 (17)	0.0098 (12)	0.0184 (14)	0.0198 (13)
C45	0.0302 (15)	0.0319 (15)	0.0326 (16)	0.0158 (12)	0.0216 (14)	0.0220 (13)
C46	0.0304 (15)	0.0266 (14)	0.0236 (15)	0.0122 (11)	0.0169 (13)	0.0131 (12)
C47	0.0200 (13)	0.0211 (13)	0.0232 (14)	0.0110 (10)	0.0140 (12)	0.0124 (11)
C48	0.0179 (13)	0.0295 (14)	0.0226 (14)	0.0106 (11)	0.0104 (12)	0.0170 (12)
C49	0.0196 (13)	0.0305 (15)	0.0313 (16)	0.0091 (11)	0.0123 (13)	0.0189 (13)
C50	0.0239 (14)	0.0335 (15)	0.0413 (18)	0.0133 (12)	0.0210 (14)	0.0256 (14)
C51	0.0345 (16)	0.0352 (16)	0.0379 (17)	0.0202 (13)	0.0267 (15)	0.0260 (14)
C52	0.0353 (16)	0.0305 (15)	0.0299 (16)	0.0162 (12)	0.0240 (14)	0.0180 (13)
C53	0.0238 (13)	0.0249 (13)	0.0242 (14)	0.0133 (11)	0.0159 (12)	0.0143 (12)
C54	0.0214 (13)	0.0165 (12)	0.0190 (13)	0.0070 (10)	0.0131 (11)	0.0090 (10)
C55	0.0169 (12)	0.0174 (12)	0.0164 (13)	0.0070 (9)	0.0072 (11)	0.0070 (10)
C56	0.0243 (13)	0.0158 (12)	0.0146 (13)	0.0092 (10)	0.0109 (11)	0.0068 (10)
C57	0.0217 (13)	0.0195 (13)	0.0189 (13)	0.0075 (10)	0.0120 (11)	0.0098 (11)
C58	0.0215 (13)	0.0168 (12)	0.0157 (13)	0.0088 (10)	0.0095 (11)	0.0077 (10)
C59	0.0221 (13)	0.0187 (12)	0.0150 (13)	0.0085 (10)	0.0102 (11)	0.0081 (10)
C60	0.0212 (13)	0.0322 (14)	0.0192 (14)	0.0121 (11)	0.0129 (12)	0.0123 (12)
C61	0.0192 (13)	0.0245 (14)	0.0176 (14)	0.0056 (10)	0.0085 (11)	0.0077 (11)
C62	0.0299 (16)	0.0292 (15)	0.0354 (17)	0.0148 (12)	0.0132 (14)	0.0143 (14)
C63	0.0185 (13)	0.0249 (13)	0.0185 (13)	0.0095 (10)	0.0095 (11)	0.0113 (11)
C64	0.0199 (13)	0.0317 (15)	0.0234 (14)	0.0091 (11)	0.0127 (12)	0.0120 (12)
C65	0.0227 (14)	0.0232 (13)	0.0261 (15)	0.0092 (11)	0.0100 (12)	0.0132 (12)
C66	0.0209 (13)	0.0207 (13)	0.0188 (13)	0.0074 (10)	0.0081 (11)	0.0079 (11)
C67	0.0213 (13)	0.0251 (14)	0.0198 (14)	0.0058 (11)	0.0074 (12)	0.0100 (11)
C68	0.0342 (17)	0.0361 (17)	0.0224 (16)	0.0016 (13)	0.0037 (14)	0.0135 (14)
C69	0.0335 (17)	0.0295 (16)	0.0305 (17)	0.0099 (13)	0.0086 (14)	0.0071 (13)
C70	0.0234 (15)	0.0407 (17)	0.0293 (17)	0.0033 (12)	0.0102 (13)	0.0133 (14)
C71	0.0235 (13)	0.0241 (13)	0.0158 (13)	0.0102 (10)	0.0113 (11)	0.0106 (11)
C72	0.0169 (12)	0.0241 (13)	0.0131 (12)	0.0074 (10)	0.0075 (11)	0.0102 (11)
C73	0.0199 (12)	0.0192 (12)	0.0141 (12)	0.0078 (10)	0.0071 (11)	0.0083 (10)
C74	0.0217 (13)	0.0173 (12)	0.0186 (13)	0.0076 (10)	0.0110 (11)	0.0113 (11)
C75	0.0198 (12)	0.0207 (13)	0.0140 (12)	0.0064 (10)	0.0089 (11)	0.0077 (10)
C76	0.0196 (13)	0.0209 (13)	0.0147 (13)	0.0067 (10)	0.0075 (11)	0.0089 (11)
C77	0.0196 (12)	0.0213 (13)	0.0177 (13)	0.0043 (10)	0.0089 (11)	0.0124 (11)
C78	0.0270 (14)	0.0198 (13)	0.0197 (14)	0.0091 (10)	0.0121 (12)	0.0121 (11)
C79	0.0349 (15)	0.0258 (14)	0.0199 (14)	0.0135 (12)	0.0166 (13)	0.0118 (12)
C80	0.0322 (15)	0.0341 (15)	0.0317 (16)	0.0208 (12)	0.0197 (14)	0.0189 (13)
C81	0.0261 (13)	0.0193 (13)	0.0165 (13)	0.0075 (10)	0.0104 (12)	0.0103 (11)
C82	0.0267 (14)	0.0201 (13)	0.0233 (14)	0.0109 (11)	0.0128 (12)	0.0099 (11)
C83	0.0339 (15)	0.0233 (14)	0.0250 (15)	0.0108 (12)	0.0121 (13)	0.0151 (12)
C84	0.0293 (14)	0.0196 (13)	0.0227 (14)	0.0081 (11)	0.0144 (12)	0.0113 (11)
C85	0.0233 (14)	0.0270 (14)	0.0203 (14)	0.0094 (11)	0.0109 (12)	0.0111 (12)
C86	0.0305 (15)	0.0325 (15)	0.0309 (16)	0.0139 (12)	0.0126 (14)	0.0202 (13)
C87	0.0290 (15)	0.0321 (15)	0.0194 (14)	0.0106 (12)	0.0100 (13)	0.0087 (12)
C88	0.0280 (15)	0.0335 (16)	0.0345 (17)	0.0072 (12)	0.0153 (14)	0.0161 (14)
C89	0.0240 (13)	0.0251 (14)	0.0303 (15)	0.0150 (11)	0.0172 (13)	0.0188 (12)
C90	0.0319 (15)	0.0404 (16)	0.0337 (17)	0.0190 (13)	0.0202 (14)	0.0280 (14)
C91	0.0382 (17)	0.0417 (17)	0.050 (2)	0.0229 (14)	0.0324 (16)	0.0357 (16)
C92	0.0360 (16)	0.0286 (15)	0.053 (2)	0.0139 (13)	0.0324 (16)	0.0254 (15)
C93	0.0311 (15)	0.0231 (14)	0.0384 (17)	0.0089 (11)	0.0216 (14)	0.0151 (13)
C94	0.0287 (14)	0.0229 (13)	0.0301 (15)	0.0136 (11)	0.0211 (13)	0.0157 (12)
C95	0.0170 (12)	0.0252 (13)	0.0218 (14)	0.0096 (10)	0.0097 (11)	0.0135 (11)
C96	0.0205 (13)	0.0270 (14)	0.0312 (16)	0.0061 (11)	0.0100 (13)	0.0173 (13)
C97	0.0260 (14)	0.0323 (15)	0.0428 (18)	0.0112 (12)	0.0202 (14)	0.0260 (14)
C98	0.0353 (16)	0.0379 (16)	0.0404 (18)	0.0209 (13)	0.0279 (15)	0.0278 (15)
C99	0.0370 (16)	0.0280 (14)	0.0289 (15)	0.0147 (12)	0.0237 (14)	0.0164 (13)
C100	0.0231 (13)	0.0221 (13)	0.0275 (15)	0.0119 (10)	0.0151 (12)	0.0157 (12)
C101	0.0184 (12)	0.0176 (12)	0.0155 (13)	0.0050 (10)	0.0091 (11)	0.0070 (10)
C102	0.0190 (12)	0.0183 (12)	0.0147 (12)	0.0083 (10)	0.0083 (11)	0.0058 (10)
C103	0.0250 (13)	0.0162 (12)	0.0153 (13)	0.0093 (10)	0.0124 (11)	0.0066 (10)
C104	0.0215 (13)	0.0205 (13)	0.0182 (13)	0.0067 (10)	0.0111 (11)	0.0092 (11)
C105	0.0204 (13)	0.0200 (13)	0.0153 (13)	0.0049 (10)	0.0089 (11)	0.0056 (10)
C106	0.0239 (13)	0.0205 (13)	0.0163 (13)	0.0072 (10)	0.0098 (11)	0.0092 (11)
C107	0.0208 (13)	0.0238 (13)	0.0181 (13)	0.0079 (10)	0.0109 (11)	0.0096 (11)
C108	0.0230 (14)	0.0321 (15)	0.0227 (14)	0.0113 (11)	0.0128 (12)	0.0160 (12)
C109	0.0339 (16)	0.0319 (15)	0.0308 (16)	0.0205 (12)	0.0187 (14)	0.0202 (13)
C110	0.0186 (13)	0.0300 (14)	0.0219 (14)	0.0073 (11)	0.0106 (12)	0.0129 (12)
C111	0.0258 (15)	0.0386 (16)	0.0274 (16)	0.0111 (12)	0.0153 (13)	0.0124 (13)
C112	0.0221 (14)	0.0263 (14)	0.0270 (15)	0.0092 (11)	0.0115 (12)	0.0132 (12)
C113	0.0194 (13)	0.0337 (15)	0.0272 (15)	0.0083 (11)	0.0118 (12)	0.0172 (13)
C114	0.0281 (15)	0.0283 (14)	0.0287 (16)	0.0115 (12)	0.0163 (13)	0.0146 (13)
C115	0.0328 (16)	0.0335 (16)	0.0362 (18)	0.0110 (13)	0.0209 (15)	0.0132 (14)
C116	0.0336 (16)	0.0283 (15)	0.0325 (17)	0.0075 (12)	0.0154 (14)	0.0130 (13)
C117	0.0343 (16)	0.0306 (15)	0.0226 (15)	0.0098 (12)	0.0101 (14)	0.0097 (13)
C118	0.0304 (14)	0.0272 (14)	0.0227 (14)	0.0153 (11)	0.0185 (13)	0.0151 (12)
C119	0.0169 (12)	0.0250 (13)	0.0159 (13)	0.0083 (10)	0.0079 (11)	0.0109 (11)
C120	0.0193 (12)	0.0192 (13)	0.0149 (13)	0.0078 (10)	0.0072 (11)	0.0073 (10)
C121	0.0163 (12)	0.0220 (13)	0.0167 (13)	0.0065 (10)	0.0061 (11)	0.0118 (11)
C122	0.0190 (12)	0.0207 (13)	0.0145 (13)	0.0072 (10)	0.0086 (11)	0.0072 (10)
C123	0.0160 (12)	0.0197 (12)	0.0161 (13)	0.0057 (10)	0.0051 (11)	0.0084 (10)
C124	0.0193 (13)	0.0243 (13)	0.0213 (14)	0.0078 (10)	0.0100 (11)	0.0152 (11)
C125	0.0321 (15)	0.0221 (13)	0.0176 (13)	0.0114 (11)	0.0117 (12)	0.0116 (11)
C126	0.0243 (14)	0.0224 (13)	0.0193 (14)	0.0069 (11)	0.0091 (12)	0.0098 (11)
C127	0.0327 (16)	0.0299 (15)	0.0340 (17)	0.0179 (13)	0.0167 (14)	0.0140 (13)
C128	0.0266 (14)	0.0200 (13)	0.0204 (14)	0.0063 (10)	0.0129 (12)	0.0083 (11)
C129	0.0249 (14)	0.0216 (13)	0.0256 (15)	0.0089 (11)	0.0119 (13)	0.0087 (12)
C130	0.0374 (16)	0.0245 (14)	0.0254 (15)	0.0107 (12)	0.0148 (14)	0.0133 (12)
C131	0.0247 (14)	0.0246 (14)	0.0247 (15)	0.0089 (11)	0.0119 (12)	0.0118 (12)
C132	0.0235 (14)	0.0247 (14)	0.0220 (14)	0.0089 (11)	0.0114 (12)	0.0098 (12)
C133	0.0415 (17)	0.0249 (15)	0.0269 (16)	0.0056 (13)	0.0136 (14)	0.0099 (13)
C134	0.0290 (16)	0.0323 (16)	0.0311 (17)	0.0079 (12)	0.0096 (14)	0.0072 (14)
C135	0.0294 (15)	0.0341 (16)	0.0214 (15)	0.0041 (12)	0.0108 (13)	0.0063 (13)
C136	0.0259 (14)	0.0217 (13)	0.0234 (14)	0.0119 (11)	0.0165 (12)	0.0123 (11)
C137	0.0305 (15)	0.0240 (14)	0.0325 (16)	0.0081 (11)	0.0199 (14)	0.0126 (12)
C138	0.0322 (16)	0.0317 (15)	0.0440 (19)	0.0153 (12)	0.0278 (15)	0.0224 (14)
C139	0.0342 (16)	0.0529 (19)	0.0401 (19)	0.0209 (14)	0.0264 (15)	0.0346 (16)
C140	0.0288 (15)	0.0521 (19)	0.0257 (16)	0.0155 (13)	0.0162 (14)	0.0235 (15)
C141	0.0224 (13)	0.0305 (14)	0.0227 (14)	0.0134 (11)	0.0141 (12)	0.0167 (12)

### Geometric parameters (Å, °)

**Table:**

O1—C8	1.377 (3)	C57—C58	1.394 (3)
O1—C13	1.425 (3)	C57—H57	0.9500
O2—C14	1.335 (3)	C58—C59	1.398 (3)
O2—C15	1.448 (3)	C58—C63	1.539 (3)
O3—C14	1.197 (3)	C59—H59	0.9500
O4—C26	1.374 (3)	C60—C61	1.506 (3)
O4—C31	1.427 (3)	C60—H60A	0.9900
O5—C32	1.339 (3)	C60—H60B	0.9900
O5—C33	1.447 (3)	C62—H62A	0.9800
O6—C32	1.188 (3)	C62—H62B	0.9800
O7—C55	1.376 (3)	C62—H62C	0.9800
O7—C60	1.426 (3)	C63—C65	1.533 (3)
O8—C61	1.344 (3)	C63—C64	1.541 (3)
O8—C62	1.445 (3)	C63—C66	1.558 (3)
O9—C61	1.192 (3)	C64—H64A	0.9800
O10—C73	1.376 (3)	C64—H64B	0.9800
O10—C78	1.425 (3)	C64—H64C	0.9800
O11—C79	1.330 (3)	C65—H65A	0.9800
O11—C80	1.447 (3)	C65—H65B	0.9800
O12—C79	1.181 (3)	C65—H65C	0.9800
O13—C102	1.375 (3)	C66—C67	1.549 (3)
O13—C107	1.428 (3)	C66—H66A	0.9900
O14—C108	1.335 (3)	C66—H66B	0.9900
O14—C109	1.449 (3)	C67—C68	1.524 (4)
O15—C108	1.184 (3)	C67—C70	1.528 (4)
O16—C120	1.383 (3)	C67—C69	1.529 (4)
O16—C125	1.417 (3)	C68—H68A	0.9800
O17—C126	1.340 (3)	C68—H68B	0.9800
O17—C127	1.443 (3)	C68—H68C	0.9800
O18—C126	1.189 (3)	C69—H69A	0.9800
N1—N2	1.334 (3)	C69—H69B	0.9800
N1—C1	1.350 (3)	C69—H69C	0.9800
N2—N3	1.336 (3)	C70—H70A	0.9800
N2—C7	1.435 (3)	C70—H70B	0.9800
N3—C6	1.350 (3)	C70—H70C	0.9800
N4—N5	1.333 (3)	C71—C72	1.516 (3)
N4—C42	1.357 (3)	C71—H71A	0.9900
N5—N6	1.332 (3)	C71—H71B	0.9900
N5—C27	1.432 (3)	C72—C77	1.391 (3)
N6—C47	1.353 (3)	C72—C73	1.400 (3)
N7—N8	1.337 (3)	C73—C74	1.384 (3)
N7—C48	1.354 (3)	C74—C75	1.394 (3)
N8—N9	1.337 (3)	C75—C76	1.389 (3)
N8—C54	1.432 (3)	C75—H75	0.9500
N9—C53	1.352 (3)	C76—C77	1.394 (3)
N10—N11	1.334 (3)	C76—C81	1.541 (3)
N10—C89	1.349 (3)	C77—H77	0.9500
N11—N12	1.331 (3)	C78—C79	1.505 (3)
N11—C74	1.437 (3)	C78—H78A	0.9900
N12—C94	1.361 (3)	C78—H78B	0.9900
N13—N14	1.334 (3)	C80—H80A	0.9800
N13—C95	1.359 (3)	C80—H80B	0.9800
N14—N15	1.331 (3)	C80—H80C	0.9800
N14—C101	1.433 (3)	C81—C82	1.526 (3)
N15—C100	1.347 (3)	C81—C83	1.545 (3)
N16—N17	1.331 (3)	C81—C84	1.547 (3)
N16—C136	1.352 (3)	C82—H82A	0.9800
N17—N18	1.338 (3)	C82—H82B	0.9800
N17—C121	1.430 (3)	C82—H82C	0.9800
N18—C141	1.352 (3)	C83—H83A	0.9800
C1—C6	1.410 (4)	C83—H83B	0.9800
C1—C2	1.420 (3)	C83—H83C	0.9800
C2—C3	1.368 (4)	C84—C85	1.541 (4)
C2—H2	0.9500	C84—H84A	0.9900
C3—C4	1.419 (4)	C84—H84B	0.9900
C3—H3	0.9500	C85—C86	1.527 (4)
C4—C5	1.361 (4)	C85—C87	1.531 (3)
C4—H4	0.9500	C85—C88	1.534 (3)
C5—C6	1.415 (3)	C86—H86A	0.9800
C5—H5	0.9500	C86—H86B	0.9800
C7—C12	1.379 (3)	C86—H86C	0.9800
C7—C8	1.392 (3)	C87—H87A	0.9800
C8—C9	1.393 (3)	C87—H87B	0.9800
C9—C10	1.395 (3)	C87—H87C	0.9800
C9—C24	1.513 (3)	C88—H88A	0.9800
C10—C11	1.397 (3)	C88—H88B	0.9800
C10—H10	0.9500	C88—H88C	0.9800
C11—C12	1.399 (3)	C89—C94	1.406 (4)
C11—C16	1.532 (3)	C89—C90	1.413 (3)
C12—H12	0.9500	C90—C91	1.360 (4)
C13—C14	1.505 (3)	C90—H90	0.9500
C13—H13A	0.9900	C91—C92	1.418 (4)
C13—H13B	0.9900	C91—H91	0.9500
C15—H15A	0.9800	C92—C93	1.372 (4)
C15—H15B	0.9800	C92—H92	0.9500
C15—H15C	0.9800	C93—C94	1.412 (3)
C16—C18	1.520 (4)	C93—H93	0.9500
C16—C17	1.540 (4)	C95—C100	1.404 (3)
C16—C19	1.584 (4)	C95—C96	1.417 (3)
C17—H17A	0.9800	C96—C97	1.365 (4)
C17—H17B	0.9800	C96—H96	0.9500
C17—H17C	0.9800	C97—C98	1.413 (4)
C18—H18A	0.9800	C97—H97	0.9500
C18—H18B	0.9800	C98—C99	1.363 (4)
C18—H18C	0.9800	C98—H98	0.9500
C19—C20	1.463 (3)	C99—C100	1.417 (3)
C19—H19A	0.9900	C99—H99	0.9500
C19—H19B	0.9900	C101—C106	1.386 (3)
C20—C21'	1.399 (3)	C101—C102	1.393 (3)
C20—C22	1.408 (3)	C102—C103	1.400 (3)
C20—C23	1.466 (3)	C103—C104	1.391 (3)
C20—C22'	1.485 (3)	C103—C118	1.512 (3)
C20—C23'	1.527 (4)	C104—C105	1.395 (3)
C20—C21	1.560 (4)	C104—H10B	0.9500
C21—H21A	0.9800	C105—C106	1.394 (3)
C21—H21B	0.9800	C105—C110	1.543 (3)
C21—H21C	0.9800	C106—H10C	0.9500
C22—H22A	0.9800	C107—C108	1.503 (3)
C22—H22B	0.9800	C107—H10D	0.9900
C22—H22C	0.9800	C107—H10E	0.9900
C23—H23A	0.9800	C109—H10F	0.9800
C23—H23B	0.9800	C109—H10G	0.9800
C23—H23C	0.9800	C109—H10H	0.9800
C21'—H21D	0.9800	C110—C112	1.518 (3)
C21'—H21E	0.9800	C110—C113	1.546 (3)
C21'—H21F	0.9800	C110—C111	1.548 (3)
C22'—H22D	0.9800	C111—H11A	0.9800
C22'—H22E	0.9800	C111—H11B	0.9800
C22'—H22F	0.9800	C111—H11C	0.9800
C23'—H23D	0.9800	C112—H11D	0.9800
C23'—H23E	0.9800	C112—H11E	0.9800
C23'—H23F	0.9800	C112—H11F	0.9800
C24—C25	1.516 (3)	C113—C114	1.555 (4)
C24—H24A	0.9900	C113—H11G	0.9900
C24—H24B	0.9900	C113—H11H	0.9900
C25—C30	1.392 (3)	C114—C115	1.526 (4)
C25—C26	1.402 (3)	C114—C117	1.534 (4)
C26—C27	1.393 (3)	C114—C116	1.535 (4)
C27—C28	1.386 (3)	C115—H11I	0.9800
C28—C29	1.389 (3)	C115—H11J	0.9800
C28—H28	0.9500	C115—H11K	0.9800
C29—C30	1.400 (3)	C116—H11L	0.9800
C29—C34	1.533 (3)	C116—H11M	0.9800
C30—H30	0.9500	C116—H11N	0.9800
C31—C32	1.510 (3)	C117—H11O	0.9800
C31—H31A	0.9900	C117—H11P	0.9800
C31—H31B	0.9900	C117—H11Q	0.9800
C33—H33A	0.9800	C118—C119	1.517 (3)
C33—H33B	0.9800	C118—H11R	0.9900
C33—H33C	0.9800	C118—H11S	0.9900
C34—C36	1.527 (3)	C119—C120	1.392 (3)
C34—C35	1.543 (3)	C119—C124	1.393 (3)
C34—C37	1.554 (3)	C120—C121	1.393 (3)
C35—H35A	0.9800	C121—C122	1.384 (3)
C35—H35B	0.9800	C122—C123	1.390 (3)
C35—H35C	0.9800	C122—H122	0.9500
C36—H36A	0.9800	C123—C124	1.395 (3)
C36—H36B	0.9800	C123—C128	1.537 (3)
C36—H36C	0.9800	C124—H124	0.9500
C37—C38	1.539 (4)	C125—C126	1.503 (3)
C37—H37A	0.9900	C125—H12D	0.9900
C37—H37B	0.9900	C125—H12E	0.9900
C38—C39	1.531 (3)	C127—H12F	0.9800
C38—C41	1.532 (4)	C127—H12G	0.9800
C38—C40	1.532 (3)	C127—H12H	0.9800
C39—H39A	0.9800	C128—C130	1.529 (3)
C39—H39B	0.9800	C128—C129	1.540 (3)
C39—H39C	0.9800	C128—C131	1.564 (4)
C40—H40A	0.9800	C129—H12I	0.9800
C40—H40B	0.9800	C129—H12J	0.9800
C40—H40C	0.9800	C129—H12K	0.9800
C41—H41A	0.9800	C130—H13C	0.9800
C41—H41B	0.9800	C130—H13D	0.9800
C41—H41C	0.9800	C130—H13E	0.9800
C42—C47	1.404 (4)	C131—C132	1.533 (3)
C42—C43	1.412 (3)	C131—H13F	0.9900
C43—C44	1.362 (4)	C131—H13G	0.9900
C43—H43	0.9500	C132—C133	1.525 (4)
C44—C45	1.420 (4)	C132—C135	1.526 (3)
C44—H44	0.9500	C132—C134	1.535 (4)
C45—C46	1.359 (3)	C133—H13H	0.9800
C45—H45	0.9500	C133—H13I	0.9800
C46—C47	1.415 (3)	C133—H13J	0.9800
C46—H46	0.9500	C134—H13K	0.9800
C48—C53	1.407 (4)	C134—H13L	0.9800
C48—C49	1.415 (3)	C134—H13M	0.9800
C49—C50	1.368 (4)	C135—H13N	0.9800
C49—H49	0.9500	C135—H13O	0.9800
C50—C51	1.414 (4)	C135—H13P	0.9800
C50—H50	0.9500	C136—C141	1.408 (4)
C51—C52	1.361 (4)	C136—C137	1.412 (3)
C51—H51	0.9500	C137—C138	1.368 (4)
C52—C53	1.417 (3)	C137—H137	0.9500
C52—H52	0.9500	C138—C139	1.421 (4)
C54—C59	1.381 (3)	C138—H138	0.9500
C54—C55	1.394 (3)	C139—C140	1.358 (4)
C55—C56	1.397 (3)	C139—H139	0.9500
C56—C57	1.396 (3)	C140—C141	1.415 (3)
C56—C71	1.509 (3)	C140—H140	0.9500
			
C8—O1—C13	115.89 (19)	H66A—C66—H66B	106.4
C14—O2—C15	116.1 (2)	C68—C67—C70	108.0 (2)
C26—O4—C31	118.18 (18)	C68—C67—C69	107.7 (2)
C32—O5—C33	116.10 (19)	C70—C67—C69	107.7 (2)
C55—O7—C60	115.42 (18)	C68—C67—C66	113.2 (2)
C61—O8—C62	115.8 (2)	C70—C67—C66	114.0 (2)
C73—O10—C78	118.62 (18)	C69—C67—C66	106.0 (2)
C79—O11—C80	116.64 (19)	C67—C68—H68A	109.5
C102—O13—C107	117.83 (18)	C67—C68—H68B	109.5
C108—O14—C109	116.40 (19)	H68A—C68—H68B	109.5
C120—O16—C125	116.33 (18)	C67—C68—H68C	109.5
C126—O17—C127	116.0 (2)	H68A—C68—H68C	109.5
N2—N1—C1	102.3 (2)	H68B—C68—H68C	109.5
N1—N2—N3	117.50 (19)	C67—C69—H69A	109.5
N1—N2—C7	120.6 (2)	C67—C69—H69B	109.5
N3—N2—C7	121.9 (2)	H69A—C69—H69B	109.5
N2—N3—C6	102.4 (2)	C67—C69—H69C	109.5
N5—N4—C42	102.41 (19)	H69A—C69—H69C	109.5
N6—N5—N4	117.38 (19)	H69B—C69—H69C	109.5
N6—N5—C27	120.12 (19)	C67—C70—H70A	109.5
N4—N5—C27	121.66 (19)	C67—C70—H70B	109.5
N5—N6—C47	102.60 (19)	H70A—C70—H70B	109.5
N8—N7—C48	102.3 (2)	C67—C70—H70C	109.5
N9—N8—N7	117.42 (19)	H70A—C70—H70C	109.5
N9—N8—C54	122.1 (2)	H70B—C70—H70C	109.5
N7—N8—C54	120.21 (19)	C56—C71—C72	114.9 (2)
N8—N9—C53	102.43 (19)	C56—C71—H71A	108.6
N11—N10—C89	102.1 (2)	C72—C71—H71A	108.6
N12—N11—N10	117.80 (19)	C56—C71—H71B	108.6
N12—N11—C74	121.9 (2)	C72—C71—H71B	108.6
N10—N11—C74	119.8 (2)	H71A—C71—H71B	107.5
N11—N12—C94	102.3 (2)	C77—C72—C73	118.7 (2)
N14—N13—C95	102.39 (19)	C77—C72—C71	119.6 (2)
N15—N14—N13	117.36 (19)	C73—C72—C71	121.6 (2)
N15—N14—C101	121.24 (19)	O10—C73—C74	123.8 (2)
N13—N14—C101	121.22 (19)	O10—C73—C72	117.6 (2)
N14—N15—C100	102.6 (2)	C74—C73—C72	118.6 (2)
N17—N16—C136	102.5 (2)	C73—C74—C75	121.6 (2)
N16—N17—N18	117.54 (19)	C73—C74—N11	122.6 (2)
N16—N17—C121	121.1 (2)	C75—C74—N11	115.7 (2)
N18—N17—C121	120.91 (19)	C76—C75—C74	120.9 (2)
N17—N18—C141	102.2 (2)	C76—C75—H75	119.6
N1—C1—C6	109.0 (2)	C74—C75—H75	119.6
N1—C1—C2	129.3 (3)	C75—C76—C77	116.7 (2)
C6—C1—C2	121.7 (2)	C75—C76—C81	122.0 (2)
C3—C2—C1	116.2 (3)	C77—C76—C81	121.1 (2)
C3—C2—H2	121.9	C72—C77—C76	123.4 (2)
C1—C2—H2	121.9	C72—C77—H77	118.3
C2—C3—C4	122.2 (2)	C76—C77—H77	118.3
C2—C3—H3	118.9	O10—C78—C79	108.83 (19)
C4—C3—H3	118.9	O10—C78—H78A	109.9
C5—C4—C3	122.4 (2)	C79—C78—H78A	109.9
C5—C4—H4	118.8	O10—C78—H78B	109.9
C3—C4—H4	118.8	C79—C78—H78B	109.9
C4—C5—C6	116.9 (3)	H78A—C78—H78B	108.3
C4—C5—H5	121.6	O12—C79—O11	124.0 (2)
C6—C5—H5	121.6	O12—C79—C78	125.5 (2)
N3—C6—C1	108.7 (2)	O11—C79—C78	110.5 (2)
N3—C6—C5	130.5 (2)	O11—C80—H80A	109.5
C1—C6—C5	120.7 (2)	O11—C80—H80B	109.5
C12—C7—C8	121.9 (2)	H80A—C80—H80B	109.5
C12—C7—N2	118.2 (2)	O11—C80—H80C	109.5
C8—C7—N2	119.8 (2)	H80A—C80—H80C	109.5
O1—C8—C7	122.1 (2)	H80B—C80—H80C	109.5
O1—C8—C9	119.1 (2)	C82—C81—C76	111.15 (19)
C7—C8—C9	118.8 (2)	C82—C81—C83	106.8 (2)
C8—C9—C10	118.8 (2)	C76—C81—C83	106.7 (2)
C8—C9—C24	119.7 (2)	C82—C81—C84	113.2 (2)
C10—C9—C24	121.3 (2)	C76—C81—C84	112.3 (2)
C9—C10—C11	122.8 (2)	C83—C81—C84	106.26 (19)
C9—C10—H10	118.6	C81—C82—H82A	109.5
C11—C10—H10	118.6	C81—C82—H82B	109.5
C10—C11—C12	117.2 (2)	H82A—C82—H82B	109.5
C10—C11—C16	122.9 (2)	C81—C82—H82C	109.5
C12—C11—C16	119.8 (2)	H82A—C82—H82C	109.5
C7—C12—C11	120.4 (2)	H82B—C82—H82C	109.5
C7—C12—H12	119.8	C81—C83—H83A	109.5
C11—C12—H12	119.8	C81—C83—H83B	109.5
O1—C13—C14	109.56 (19)	H83A—C83—H83B	109.5
O1—C13—H13A	109.8	C81—C83—H83C	109.5
C14—C13—H13A	109.8	H83A—C83—H83C	109.5
O1—C13—H13B	109.8	H83B—C83—H83C	109.5
C14—C13—H13B	109.8	C85—C84—C81	122.9 (2)
H13A—C13—H13B	108.2	C85—C84—H84A	106.6
O3—C14—O2	124.4 (2)	C81—C84—H84A	106.6
O3—C14—C13	126.0 (2)	C85—C84—H84B	106.6
O2—C14—C13	109.5 (2)	C81—C84—H84B	106.6
O2—C15—H15A	109.5	H84A—C84—H84B	106.6
O2—C15—H15B	109.5	C86—C85—C87	110.5 (2)
H15A—C15—H15B	109.5	C86—C85—C88	107.6 (2)
O2—C15—H15C	109.5	C87—C85—C88	106.7 (2)
H15A—C15—H15C	109.5	C86—C85—C84	112.8 (2)
H15B—C15—H15C	109.5	C87—C85—C84	111.8 (2)
C18—C16—C11	110.3 (2)	C88—C85—C84	107.1 (2)
C18—C16—C17	107.8 (3)	C85—C86—H86A	109.5
C11—C16—C17	109.6 (2)	C85—C86—H86B	109.5
C18—C16—C19	112.5 (3)	H86A—C86—H86B	109.5
C11—C16—C19	106.4 (2)	C85—C86—H86C	109.5
C17—C16—C19	110.2 (2)	H86A—C86—H86C	109.5
C16—C17—H17A	109.5	H86B—C86—H86C	109.5
C16—C17—H17B	109.5	C85—C87—H87A	109.5
H17A—C17—H17B	109.5	C85—C87—H87B	109.5
C16—C17—H17C	109.5	H87A—C87—H87B	109.5
H17A—C17—H17C	109.5	C85—C87—H87C	109.5
H17B—C17—H17C	109.5	H87A—C87—H87C	109.5
C16—C18—H18A	109.5	H87B—C87—H87C	109.5
C16—C18—H18B	109.5	C85—C88—H88A	109.5
H18A—C18—H18B	109.5	C85—C88—H88B	109.5
C16—C18—H18C	109.5	H88A—C88—H88B	109.5
H18A—C18—H18C	109.5	C85—C88—H88C	109.5
H18B—C18—H18C	109.5	H88A—C88—H88C	109.5
C20—C19—C16	128.6 (2)	H88B—C88—H88C	109.5
C20—C19—H19A	105.1	N10—C89—C94	109.4 (2)
C16—C19—H19A	105.1	N10—C89—C90	129.3 (3)
C20—C19—H19B	105.1	C94—C89—C90	121.2 (2)
C16—C19—H19B	105.1	C91—C90—C89	116.7 (3)
H19A—C19—H19B	105.9	C91—C90—H90	121.7
C22—C20—C19	118.6 (3)	C89—C90—H90	121.7
C22—C20—C23	114.0 (3)	C90—C91—C92	122.2 (3)
C19—C20—C23	110.2 (3)	C90—C91—H91	118.9
C21'—C20—C22'	113.0 (3)	C92—C91—H91	118.9
C19—C20—C22'	109.9 (3)	C93—C92—C91	122.4 (2)
C21'—C20—C23'	108.2 (3)	C93—C92—H92	118.8
C19—C20—C23'	110.3 (3)	C91—C92—H92	118.8
C22'—C20—C23'	101.6 (3)	C92—C93—C94	116.0 (3)
C22—C20—C21	106.2 (3)	C92—C93—H93	122.0
C19—C20—C21	103.8 (2)	C94—C93—H93	122.0
C23—C20—C21	102.1 (3)	N12—C94—C89	108.3 (2)
C20—C21—H21A	109.5	N12—C94—C93	130.1 (3)
C20—C21—H21B	109.5	C89—C94—C93	121.5 (2)
C20—C21—H21C	109.5	N13—C95—C100	108.5 (2)
C20—C22—H22A	109.5	N13—C95—C96	130.0 (2)
C20—C22—H22B	109.5	C100—C95—C96	121.4 (2)
C20—C22—H22C	109.5	C97—C96—C95	116.6 (3)
C20—C23—H23A	109.5	C97—C96—H96	121.7
C20—C23—H23B	109.5	C95—C96—H96	121.7
C20—C23—H23C	109.5	C96—C97—C98	122.0 (2)
C20—C21'—H21D	109.5	C96—C97—H97	119.0
C20—C21'—H21E	109.5	C98—C97—H97	119.0
H21D—C21'—H21E	109.5	C99—C98—C97	122.4 (2)
C20—C21'—H21F	109.5	C99—C98—H98	118.8
H21D—C21'—H21F	109.5	C97—C98—H98	118.8
H21E—C21'—H21F	109.5	C98—C99—C100	116.9 (3)
C20—C22'—H22D	109.5	C98—C99—H99	121.6
C20—C22'—H22E	109.5	C100—C99—H99	121.6
H22D—C22'—H22E	109.5	N15—C100—C95	109.1 (2)
C20—C22'—H22F	109.5	N15—C100—C99	130.1 (2)
H22D—C22'—H22F	109.5	C95—C100—C99	120.7 (2)
H22E—C22'—H22F	109.5	C106—C101—C102	121.3 (2)
C20—C23'—H23D	109.5	C106—C101—N14	117.2 (2)
C20—C23'—H23E	109.5	C102—C101—N14	121.4 (2)
H23D—C23'—H23E	109.5	O13—C102—C101	123.1 (2)
C20—C23'—H23F	109.5	O13—C102—C103	118.2 (2)
H23D—C23'—H23F	109.5	C101—C102—C103	118.7 (2)
H23E—C23'—H23F	109.5	C104—C103—C102	118.7 (2)
C9—C24—C25	115.3 (2)	C104—C103—C118	119.6 (2)
C9—C24—H24A	108.4	C102—C103—C118	121.7 (2)
C25—C24—H24A	108.4	C103—C104—C105	123.5 (2)
C9—C24—H24B	108.4	C103—C104—H10B	118.3
C25—C24—H24B	108.4	C105—C104—H10B	118.3
H24A—C24—H24B	107.5	C106—C105—C104	116.5 (2)
C30—C25—C26	119.0 (2)	C106—C105—C110	122.5 (2)
C30—C25—C24	120.2 (2)	C104—C105—C110	120.7 (2)
C26—C25—C24	120.7 (2)	C101—C106—C105	121.3 (2)
O4—C26—C27	124.0 (2)	C101—C106—H10C	119.4
O4—C26—C25	117.7 (2)	C105—C106—H10C	119.4
C27—C26—C25	118.3 (2)	O13—C107—C108	108.92 (19)
C28—C27—C26	121.5 (2)	O13—C107—H10D	109.9
C28—C27—N5	116.2 (2)	C108—C107—H10D	109.9
C26—C27—N5	122.3 (2)	O13—C107—H10E	109.9
C27—C28—C29	121.4 (2)	C108—C107—H10E	109.9
C27—C28—H28	119.3	H10D—C107—H10E	108.3
C29—C28—H28	119.3	O15—C108—O14	123.7 (2)
C28—C29—C30	116.5 (2)	O15—C108—C107	126.2 (2)
C28—C29—C34	122.5 (2)	O14—C108—C107	110.1 (2)
C30—C29—C34	120.8 (2)	O14—C109—H10F	109.5
C25—C30—C29	123.2 (2)	O14—C109—H10G	109.5
C25—C30—H30	118.4	H10F—C109—H10G	109.5
C29—C30—H30	118.4	O14—C109—H10H	109.5
O4—C31—C32	108.40 (19)	H10F—C109—H10H	109.5
O4—C31—H31A	110.0	H10G—C109—H10H	109.5
C32—C31—H31A	110.0	C112—C110—C105	111.5 (2)
O4—C31—H31B	110.0	C112—C110—C113	112.7 (2)
C32—C31—H31B	110.0	C105—C110—C113	112.6 (2)
H31A—C31—H31B	108.4	C112—C110—C111	106.7 (2)
O6—C32—O5	124.1 (2)	C105—C110—C111	106.8 (2)
O6—C32—C31	126.2 (2)	C113—C110—C111	106.0 (2)
O5—C32—C31	109.7 (2)	C110—C111—H11A	109.5
O5—C33—H33A	109.5	C110—C111—H11B	109.5
O5—C33—H33B	109.5	H11A—C111—H11B	109.5
H33A—C33—H33B	109.5	C110—C111—H11C	109.5
O5—C33—H33C	109.5	H11A—C111—H11C	109.5
H33A—C33—H33C	109.5	H11B—C111—H11C	109.5
H33B—C33—H33C	109.5	C110—C112—H11D	109.5
C36—C34—C29	111.0 (2)	C110—C112—H11E	109.5
C36—C34—C35	106.5 (2)	H11D—C112—H11E	109.5
C29—C34—C35	107.2 (2)	C110—C112—H11F	109.5
C36—C34—C37	112.8 (2)	H11D—C112—H11F	109.5
C29—C34—C37	112.7 (2)	H11E—C112—H11F	109.5
C35—C34—C37	106.1 (2)	C110—C113—C114	122.9 (2)
C34—C35—H35A	109.5	C110—C113—H11G	106.6
C34—C35—H35B	109.5	C114—C113—H11G	106.6
H35A—C35—H35B	109.5	C110—C113—H11H	106.6
C34—C35—H35C	109.5	C114—C113—H11H	106.6
H35A—C35—H35C	109.5	H11G—C113—H11H	106.6
H35B—C35—H35C	109.5	C115—C114—C117	111.3 (2)
C34—C36—H36A	109.5	C115—C114—C116	108.5 (2)
C34—C36—H36B	109.5	C117—C114—C116	107.1 (2)
H36A—C36—H36B	109.5	C115—C114—C113	111.3 (2)
C34—C36—H36C	109.5	C117—C114—C113	111.6 (2)
H36A—C36—H36C	109.5	C116—C114—C113	106.7 (2)
H36B—C36—H36C	109.5	C114—C115—H11I	109.5
C38—C37—C34	123.7 (2)	C114—C115—H11J	109.5
C38—C37—H37A	106.4	H11I—C115—H11J	109.5
C34—C37—H37A	106.4	C114—C115—H11K	109.5
C38—C37—H37B	106.4	H11I—C115—H11K	109.5
C34—C37—H37B	106.4	H11J—C115—H11K	109.5
H37A—C37—H37B	106.5	C114—C116—H11L	109.5
C39—C38—C41	107.1 (2)	C114—C116—H11M	109.5
C39—C38—C40	107.6 (2)	H11L—C116—H11M	109.5
C41—C38—C40	110.3 (2)	C114—C116—H11N	109.5
C39—C38—C37	107.3 (2)	H11L—C116—H11N	109.5
C41—C38—C37	112.1 (2)	H11M—C116—H11N	109.5
C40—C38—C37	112.2 (2)	C114—C117—H11O	109.5
C38—C39—H39A	109.5	C114—C117—H11P	109.5
C38—C39—H39B	109.5	H11O—C117—H11P	109.5
H39A—C39—H39B	109.5	C114—C117—H11Q	109.5
C38—C39—H39C	109.5	H11O—C117—H11Q	109.5
H39A—C39—H39C	109.5	H11P—C117—H11Q	109.5
H39B—C39—H39C	109.5	C103—C118—C119	114.9 (2)
C38—C40—H40A	109.5	C103—C118—H11R	108.5
C38—C40—H40B	109.5	C119—C118—H11R	108.5
H40A—C40—H40B	109.5	C103—C118—H11S	108.5
C38—C40—H40C	109.5	C119—C118—H11S	108.5
H40A—C40—H40C	109.5	H11R—C118—H11S	107.5
H40B—C40—H40C	109.5	C120—C119—C124	118.7 (2)
C38—C41—H41A	109.5	C120—C119—C118	119.5 (2)
C38—C41—H41B	109.5	C124—C119—C118	121.7 (2)
H41A—C41—H41B	109.5	O16—C120—C119	118.4 (2)
C38—C41—H41C	109.5	O16—C120—C121	122.7 (2)
H41A—C41—H41C	109.5	C119—C120—C121	118.9 (2)
H41B—C41—H41C	109.5	C122—C121—C120	121.5 (2)
N4—C42—C47	108.8 (2)	C122—C121—N17	117.5 (2)
N4—C42—C43	130.5 (2)	C120—C121—N17	121.0 (2)
C47—C42—C43	120.7 (2)	C121—C122—C123	120.7 (2)
C44—C43—C42	117.0 (3)	C121—C122—H122	119.6
C44—C43—H43	121.5	C123—C122—H122	119.6
C42—C43—H43	121.5	C122—C123—C124	117.2 (2)
C43—C44—C45	121.9 (2)	C122—C123—C128	120.3 (2)
C43—C44—H44	119.1	C124—C123—C128	122.5 (2)
C45—C44—H44	119.1	C119—C124—C123	123.0 (2)
C46—C45—C44	122.5 (2)	C119—C124—H124	118.5
C46—C45—H45	118.8	C123—C124—H124	118.5
C44—C45—H45	118.8	O16—C125—C126	109.18 (19)
C45—C46—C47	116.2 (2)	O16—C125—H12D	109.8
C45—C46—H46	121.9	C126—C125—H12D	109.8
C47—C46—H46	121.9	O16—C125—H12E	109.8
N6—C47—C42	108.8 (2)	C126—C125—H12E	109.8
N6—C47—C46	129.4 (2)	H12D—C125—H12E	108.3
C42—C47—C46	121.7 (2)	O18—C126—O17	124.0 (2)
N7—C48—C53	109.0 (2)	O18—C126—C125	126.5 (2)
N7—C48—C49	129.2 (2)	O17—C126—C125	109.5 (2)
C53—C48—C49	121.7 (2)	O17—C127—H12F	109.5
C50—C49—C48	116.7 (3)	O17—C127—H12G	109.5
C50—C49—H49	121.6	H12F—C127—H12G	109.5
C48—C49—H49	121.6	O17—C127—H12H	109.5
C49—C50—C51	121.4 (2)	H12F—C127—H12H	109.5
C49—C50—H50	119.3	H12G—C127—H12H	109.5
C51—C50—H50	119.3	C130—C128—C123	109.4 (2)
C52—C51—C50	122.9 (2)	C130—C128—C129	107.8 (2)
C52—C51—H51	118.5	C123—C128—C129	109.54 (19)
C50—C51—H51	118.5	C130—C128—C131	113.5 (2)
C51—C52—C53	116.9 (3)	C123—C128—C131	104.0 (2)
C51—C52—H52	121.6	C129—C128—C131	112.5 (2)
C53—C52—H52	121.6	C128—C129—H12I	109.5
N9—C53—C48	108.9 (2)	C128—C129—H12J	109.5
N9—C53—C52	130.6 (2)	H12I—C129—H12J	109.5
C48—C53—C52	120.4 (2)	C128—C129—H12K	109.5
C59—C54—C55	121.5 (2)	H12I—C129—H12K	109.5
C59—C54—N8	118.4 (2)	H12J—C129—H12K	109.5
C55—C54—N8	120.1 (2)	C128—C130—H13C	109.5
O7—C55—C54	121.9 (2)	C128—C130—H13D	109.5
O7—C55—C56	118.8 (2)	H13C—C130—H13D	109.5
C54—C55—C56	119.2 (2)	C128—C130—H13E	109.5
C57—C56—C55	118.2 (2)	H13C—C130—H13E	109.5
C57—C56—C71	121.9 (2)	H13D—C130—H13E	109.5
C55—C56—C71	119.8 (2)	C132—C131—C128	124.7 (2)
C58—C57—C56	123.3 (2)	C132—C131—H13F	106.2
C58—C57—H57	118.4	C128—C131—H13F	106.2
C56—C57—H57	118.4	C132—C131—H13G	106.2
C57—C58—C59	117.1 (2)	C128—C131—H13G	106.2
C57—C58—C63	122.5 (2)	H13F—C131—H13G	106.3
C59—C58—C63	120.3 (2)	C133—C132—C135	108.1 (2)
C54—C59—C58	120.6 (2)	C133—C132—C131	113.9 (2)
C54—C59—H59	119.7	C135—C132—C131	113.6 (2)
C58—C59—H59	119.7	C133—C132—C134	107.2 (2)
O7—C60—C61	109.27 (19)	C135—C132—C134	107.4 (2)
O7—C60—H60A	109.8	C131—C132—C134	106.2 (2)
C61—C60—H60A	109.8	C132—C133—H13H	109.5
O7—C60—H60B	109.8	C132—C133—H13I	109.5
C61—C60—H60B	109.8	H13H—C133—H13I	109.5
H60A—C60—H60B	108.3	C132—C133—H13J	109.5
O9—C61—O8	124.4 (2)	H13H—C133—H13J	109.5
O9—C61—C60	126.9 (2)	H13I—C133—H13J	109.5
O8—C61—C60	108.7 (2)	C132—C134—H13K	109.5
O8—C62—H62A	109.5	C132—C134—H13L	109.5
O8—C62—H62B	109.5	H13K—C134—H13L	109.5
H62A—C62—H62B	109.5	C132—C134—H13M	109.5
O8—C62—H62C	109.5	H13K—C134—H13M	109.5
H62A—C62—H62C	109.5	H13L—C134—H13M	109.5
H62B—C62—H62C	109.5	C132—C135—H13N	109.5
C65—C63—C58	110.04 (19)	C132—C135—H13O	109.5
C65—C63—C64	107.2 (2)	H13N—C135—H13O	109.5
C58—C63—C64	109.2 (2)	C132—C135—H13P	109.5
C65—C63—C66	113.2 (2)	H13N—C135—H13P	109.5
C58—C63—C66	104.76 (18)	H13O—C135—H13P	109.5
C64—C63—C66	112.4 (2)	N16—C136—C141	108.7 (2)
C63—C64—H64A	109.5	N16—C136—C137	130.0 (2)
C63—C64—H64B	109.5	C141—C136—C137	121.2 (2)
H64A—C64—H64B	109.5	C138—C137—C136	116.8 (3)
C63—C64—H64C	109.5	C138—C137—H137	121.6
H64A—C64—H64C	109.5	C136—C137—H137	121.6
H64B—C64—H64C	109.5	C137—C138—C139	121.6 (2)
C63—C65—H65A	109.5	C137—C138—H138	119.2
C63—C65—H65B	109.5	C139—C138—H138	119.2
H65A—C65—H65B	109.5	C140—C139—C138	122.7 (3)
C63—C65—H65C	109.5	C140—C139—H139	118.7
H65A—C65—H65C	109.5	C138—C139—H139	118.7
H65B—C65—H65C	109.5	C139—C140—C141	116.6 (3)
C67—C66—C63	124.4 (2)	C139—C140—H140	121.7
C67—C66—H66A	106.2	C141—C140—H140	121.7
C63—C66—H66A	106.2	N18—C141—C136	109.0 (2)
C67—C66—H66B	106.2	N18—C141—C140	129.8 (2)
C63—C66—H66B	106.2	C136—C141—C140	121.1 (2)
			
C1—N1—N2—N3	−0.5 (3)	C59—C58—C63—C65	34.2 (3)
C1—N1—N2—C7	−177.5 (2)	C57—C58—C63—C64	−30.0 (3)
N1—N2—N3—C6	0.2 (3)	C59—C58—C63—C64	151.7 (2)
C7—N2—N3—C6	177.1 (2)	C57—C58—C63—C66	90.6 (3)
C42—N4—N5—N6	−0.8 (3)	C59—C58—C63—C66	−87.7 (3)
C42—N4—N5—C27	−170.2 (2)	C65—C63—C66—C67	51.6 (3)
N4—N5—N6—C47	0.8 (3)	C58—C63—C66—C67	171.5 (2)
C27—N5—N6—C47	170.5 (2)	C64—C63—C66—C67	−70.0 (3)
C48—N7—N8—N9	0.5 (3)	C63—C66—C67—C68	−69.6 (3)
C48—N7—N8—C54	174.6 (2)	C63—C66—C67—C70	54.3 (3)
N7—N8—N9—C53	−0.8 (3)	C63—C66—C67—C69	172.5 (2)
C54—N8—N9—C53	−174.8 (2)	C57—C56—C71—C72	44.2 (3)
C89—N10—N11—N12	−0.8 (3)	C55—C56—C71—C72	−138.9 (2)
C89—N10—N11—C74	−173.0 (2)	C56—C71—C72—C77	61.6 (3)
N10—N11—N12—C94	1.1 (3)	C56—C71—C72—C73	−121.5 (2)
C74—N11—N12—C94	173.1 (2)	C78—O10—C73—C74	52.5 (3)
C95—N13—N14—N15	0.8 (3)	C78—O10—C73—C72	−130.5 (2)
C95—N13—N14—C101	176.0 (2)	C77—C72—C73—O10	−174.7 (2)
N13—N14—N15—C100	−0.6 (3)	C71—C72—C73—O10	8.4 (3)
C101—N14—N15—C100	−175.8 (2)	C77—C72—C73—C74	2.5 (3)
C136—N16—N17—N18	0.7 (3)	C71—C72—C73—C74	−174.4 (2)
C136—N16—N17—C121	173.4 (2)	O10—C73—C74—C75	175.2 (2)
N16—N17—N18—C141	−0.6 (3)	C72—C73—C74—C75	−1.8 (4)
C121—N17—N18—C141	−173.3 (2)	O10—C73—C74—N11	−2.5 (4)
N2—N1—C1—C6	0.6 (2)	C72—C73—C74—N11	−179.5 (2)
N2—N1—C1—C2	178.2 (2)	N12—N11—C74—C73	55.4 (3)
N1—C1—C2—C3	−176.8 (2)	N10—N11—C74—C73	−132.7 (2)
C6—C1—C2—C3	0.6 (4)	N12—N11—C74—C75	−122.5 (2)
C1—C2—C3—C4	0.2 (4)	N10—N11—C74—C75	49.4 (3)
C2—C3—C4—C5	−0.7 (4)	C73—C74—C75—C76	−0.6 (4)
C3—C4—C5—C6	0.4 (4)	N11—C74—C75—C76	177.3 (2)
N2—N3—C6—C1	0.2 (2)	C74—C75—C76—C77	2.1 (4)
N2—N3—C6—C5	−177.0 (3)	C74—C75—C76—C81	−172.5 (2)
N1—C1—C6—N3	−0.6 (3)	C73—C72—C77—C76	−1.0 (4)
C2—C1—C6—N3	−178.4 (2)	C71—C72—C77—C76	176.0 (2)
N1—C1—C6—C5	177.0 (2)	C75—C76—C77—C72	−1.3 (4)
C2—C1—C6—C5	−0.8 (4)	C81—C76—C77—C72	173.3 (2)
C4—C5—C6—N3	177.3 (2)	C73—O10—C78—C79	132.3 (2)
C4—C5—C6—C1	0.3 (4)	C80—O11—C79—O12	0.1 (4)
N1—N2—C7—C12	124.4 (2)	C80—O11—C79—C78	−179.7 (2)
N3—N2—C7—C12	−52.4 (3)	O10—C78—C79—O12	−4.2 (4)
N1—N2—C7—C8	−53.8 (3)	O10—C78—C79—O11	175.5 (2)
N3—N2—C7—C8	129.3 (2)	C75—C76—C81—C82	3.8 (3)
C13—O1—C8—C7	−61.1 (3)	C77—C76—C81—C82	−170.6 (2)
C13—O1—C8—C9	120.5 (2)	C75—C76—C81—C83	119.8 (2)
C12—C7—C8—O1	179.1 (2)	C77—C76—C81—C83	−54.5 (3)
N2—C7—C8—O1	−2.8 (3)	C75—C76—C81—C84	−124.1 (2)
C12—C7—C8—C9	−2.6 (4)	C77—C76—C81—C84	61.5 (3)
N2—C7—C8—C9	175.6 (2)	C82—C81—C84—C85	−47.1 (3)
O1—C8—C9—C10	179.0 (2)	C76—C81—C84—C85	79.7 (3)
C7—C8—C9—C10	0.7 (3)	C83—C81—C84—C85	−164.0 (2)
O1—C8—C9—C24	−5.3 (3)	C81—C84—C85—C86	−48.2 (3)
C7—C8—C9—C24	176.3 (2)	C81—C84—C85—C87	77.0 (3)
C8—C9—C10—C11	1.0 (4)	C81—C84—C85—C88	−166.4 (2)
C24—C9—C10—C11	−174.6 (2)	N11—N10—C89—C94	0.1 (2)
C9—C10—C11—C12	−0.7 (4)	N11—N10—C89—C90	176.1 (3)
C9—C10—C11—C16	176.5 (2)	N10—C89—C90—C91	−176.9 (2)
C8—C7—C12—C11	2.9 (4)	C94—C89—C90—C91	−1.3 (4)
N2—C7—C12—C11	−175.3 (2)	C89—C90—C91—C92	−0.6 (4)
C10—C11—C12—C7	−1.2 (4)	C90—C91—C92—C93	1.9 (4)
C16—C11—C12—C7	−178.5 (2)	C91—C92—C93—C94	−1.1 (4)
C8—O1—C13—C14	−123.9 (2)	N11—N12—C94—C89	−0.9 (3)
C15—O2—C14—O3	−0.6 (4)	N11—N12—C94—C93	−178.6 (2)
C15—O2—C14—C13	177.3 (2)	N10—C89—C94—N12	0.6 (3)
O1—C13—C14—O3	−5.6 (4)	C90—C89—C94—N12	−175.8 (2)
O1—C13—C14—O2	176.50 (19)	N10—C89—C94—C93	178.5 (2)
C10—C11—C16—C18	24.8 (4)	C90—C89—C94—C93	2.1 (4)
C12—C11—C16—C18	−158.0 (3)	C92—C93—C94—N12	176.6 (3)
C10—C11—C16—C17	143.4 (2)	C92—C93—C94—C89	−0.9 (4)
C12—C11—C16—C17	−39.5 (3)	N14—N13—C95—C100	−0.6 (2)
C10—C11—C16—C19	−97.5 (3)	N14—N13—C95—C96	−178.1 (2)
C12—C11—C16—C19	79.7 (3)	N13—C95—C96—C97	176.4 (2)
C18—C16—C19—C20	56.7 (4)	C100—C95—C96—C97	−0.7 (4)
C11—C16—C19—C20	177.6 (2)	C95—C96—C97—C98	−0.2 (4)
C17—C16—C19—C20	−63.7 (3)	C96—C97—C98—C99	0.9 (4)
C16—C19—C20—C21'	−162.5 (3)	C97—C98—C99—C100	−0.6 (4)
C16—C19—C20—C22	41.5 (4)	N14—N15—C100—C95	0.1 (3)
C16—C19—C20—C23	−92.5 (4)	N14—N15—C100—C99	176.8 (3)
C16—C19—C20—C22'	70.2 (4)	N13—C95—C100—N15	0.3 (3)
C16—C19—C20—C23'	−41.0 (4)	C96—C95—C100—N15	178.0 (2)
C16—C19—C20—C21	158.9 (3)	N13—C95—C100—C99	−176.7 (2)
C8—C9—C24—C25	134.9 (2)	C96—C95—C100—C99	1.0 (4)
C10—C9—C24—C25	−49.6 (3)	C98—C99—C100—N15	−176.6 (2)
C9—C24—C25—C30	−58.5 (3)	C98—C99—C100—C95	−0.3 (4)
C9—C24—C25—C26	125.5 (2)	N15—N14—C101—C106	56.3 (3)
C31—O4—C26—C27	−56.1 (3)	N13—N14—C101—C106	−118.7 (2)
C31—O4—C26—C25	127.1 (2)	N15—N14—C101—C102	−125.3 (2)
C30—C25—C26—O4	175.1 (2)	N13—N14—C101—C102	59.7 (3)
C24—C25—C26—O4	−8.9 (3)	C107—O13—C102—C101	56.3 (3)
C30—C25—C26—C27	−1.9 (3)	C107—O13—C102—C103	−127.3 (2)
C24—C25—C26—C27	174.1 (2)	C106—C101—C102—O13	175.4 (2)
O4—C26—C27—C28	−175.5 (2)	N14—C101—C102—O13	−2.9 (3)
C25—C26—C27—C28	1.3 (3)	C106—C101—C102—C103	−1.0 (3)
O4—C26—C27—N5	2.4 (4)	N14—C101—C102—C103	−179.3 (2)
C25—C26—C27—N5	179.2 (2)	O13—C102—C103—C104	−174.6 (2)
N6—N5—C27—C28	−42.9 (3)	C101—C102—C103—C104	2.0 (3)
N4—N5—C27—C28	126.3 (2)	O13—C102—C103—C118	8.2 (3)
N6—N5—C27—C26	139.1 (2)	C101—C102—C103—C118	−175.2 (2)
N4—N5—C27—C26	−51.7 (3)	C102—C103—C104—C105	−0.8 (4)
C26—C27—C28—C29	1.3 (4)	C118—C103—C104—C105	176.5 (2)
N5—C27—C28—C29	−176.8 (2)	C103—C104—C105—C106	−1.5 (4)
C27—C28—C29—C30	−3.1 (3)	C103—C104—C105—C110	172.0 (2)
C27—C28—C29—C34	172.1 (2)	C102—C101—C106—C105	−1.4 (4)
C26—C25—C30—C29	0.0 (4)	N14—C101—C106—C105	177.1 (2)
C24—C25—C30—C29	−176.1 (2)	C104—C105—C106—C101	2.5 (3)
C28—C29—C30—C25	2.5 (4)	C110—C105—C106—C101	−170.9 (2)
C34—C29—C30—C25	−172.8 (2)	C102—O13—C107—C108	124.6 (2)
C26—O4—C31—C32	−138.1 (2)	C109—O14—C108—O15	1.2 (4)
C33—O5—C32—O6	−1.7 (4)	C109—O14—C108—C107	−178.2 (2)
C33—O5—C32—C31	178.2 (2)	O13—C107—C108—O15	−2.0 (4)
O4—C31—C32—O6	2.8 (4)	O13—C107—C108—O14	177.4 (2)
O4—C31—C32—O5	−177.1 (2)	C106—C105—C110—C112	1.9 (3)
C28—C29—C34—C36	−8.0 (3)	C104—C105—C110—C112	−171.3 (2)
C30—C29—C34—C36	167.0 (2)	C106—C105—C110—C113	−126.0 (2)
C28—C29—C34—C35	−124.0 (3)	C104—C105—C110—C113	60.8 (3)
C30—C29—C34—C35	51.0 (3)	C106—C105—C110—C111	118.0 (3)
C28—C29—C34—C37	119.7 (2)	C104—C105—C110—C111	−55.1 (3)
C30—C29—C34—C37	−65.3 (3)	C112—C110—C113—C114	−47.2 (3)
C36—C34—C37—C38	45.9 (3)	C105—C110—C113—C114	80.1 (3)
C29—C34—C37—C38	−80.8 (3)	C111—C110—C113—C114	−163.5 (2)
C35—C34—C37—C38	162.2 (2)	C110—C113—C114—C115	−47.4 (3)
C34—C37—C38—C39	167.0 (2)	C110—C113—C114—C117	77.7 (3)
C34—C37—C38—C41	−75.7 (3)	C110—C113—C114—C116	−165.7 (2)
C34—C37—C38—C40	49.1 (3)	C104—C103—C118—C119	61.6 (3)
N5—N4—C42—C47	0.4 (3)	C102—C103—C118—C119	−121.2 (2)
N5—N4—C42—C43	178.3 (3)	C103—C118—C119—C120	−139.1 (2)
N4—C42—C43—C44	−177.0 (3)	C103—C118—C119—C124	44.2 (3)
C47—C42—C43—C44	0.8 (4)	C125—O16—C120—C119	−118.4 (2)
C42—C43—C44—C45	0.1 (4)	C125—O16—C120—C121	63.2 (3)
C43—C44—C45—C46	−0.7 (4)	C124—C119—C120—O16	−178.1 (2)
C44—C45—C46—C47	0.2 (4)	C118—C119—C120—O16	5.1 (3)
N5—N6—C47—C42	−0.5 (2)	C124—C119—C120—C121	0.4 (4)
N5—N6—C47—C46	−177.0 (2)	C118—C119—C120—C121	−176.5 (2)
N4—C42—C47—N6	0.1 (3)	O16—C120—C121—C122	179.6 (2)
C43—C42—C47—N6	−178.1 (2)	C119—C120—C121—C122	1.2 (4)
N4—C42—C47—C46	176.9 (2)	O16—C120—C121—N17	2.3 (4)
C43—C42—C47—C46	−1.3 (4)	C119—C120—C121—N17	−176.1 (2)
C45—C46—C47—N6	176.8 (2)	N16—N17—C121—C122	−128.7 (2)
C45—C46—C47—C42	0.7 (4)	N18—N17—C121—C122	43.7 (3)
N8—N7—C48—C53	0.1 (3)	N16—N17—C121—C120	48.7 (3)
N8—N7—C48—C49	−177.0 (2)	N18—N17—C121—C120	−138.9 (2)
N7—C48—C49—C50	176.2 (2)	C120—C121—C122—C123	−2.2 (4)
C53—C48—C49—C50	−0.5 (4)	N17—C121—C122—C123	175.3 (2)
C48—C49—C50—C51	0.4 (4)	C121—C122—C123—C124	1.5 (4)
C49—C50—C51—C52	−0.1 (4)	C121—C122—C123—C128	178.4 (2)
C50—C51—C52—C53	−0.2 (4)	C120—C119—C124—C123	−1.0 (4)
N8—N9—C53—C48	0.7 (3)	C118—C119—C124—C123	175.7 (2)
N8—N9—C53—C52	176.7 (3)	C122—C123—C124—C119	0.1 (4)
N7—C48—C53—N9	−0.5 (3)	C128—C123—C124—C119	−176.8 (2)
C49—C48—C53—N9	176.8 (2)	C120—O16—C125—C126	135.0 (2)
N7—C48—C53—C52	−177.0 (2)	C127—O17—C126—O18	1.3 (4)
C49—C48—C53—C52	0.3 (4)	C127—O17—C126—C125	−178.3 (2)
C51—C52—C53—N9	−175.6 (3)	O16—C125—C126—O18	−0.7 (4)
C51—C52—C53—C48	0.1 (4)	O16—C125—C126—O17	178.9 (2)
N9—N8—C54—C59	48.5 (3)	C122—C123—C128—C130	152.5 (2)
N7—N8—C54—C59	−125.3 (2)	C124—C123—C128—C130	−30.7 (3)
N9—N8—C54—C55	−133.2 (2)	C122—C123—C128—C129	34.6 (3)
N7—N8—C54—C55	53.0 (3)	C124—C123—C128—C129	−148.6 (2)
C60—O7—C55—C54	66.2 (3)	C122—C123—C128—C131	−85.9 (3)
C60—O7—C55—C56	−115.7 (2)	C124—C123—C128—C131	90.9 (3)
C59—C54—C55—O7	−179.6 (2)	C130—C128—C131—C132	−68.4 (3)
N8—C54—C55—O7	2.2 (3)	C123—C128—C131—C132	172.8 (2)
C59—C54—C55—C56	2.3 (3)	C129—C128—C131—C132	54.3 (3)
N8—C54—C55—C56	−175.9 (2)	C128—C131—C132—C133	55.0 (3)
O7—C55—C56—C57	−178.5 (2)	C128—C131—C132—C135	−69.4 (3)
C54—C55—C56—C57	−0.3 (3)	C128—C131—C132—C134	172.8 (2)
O7—C55—C56—C71	4.4 (3)	N17—N16—C136—C141	−0.5 (3)
C54—C55—C56—C71	−177.4 (2)	N17—N16—C136—C137	−177.7 (2)
C55—C56—C57—C58	−1.3 (4)	N16—C136—C137—C138	177.5 (3)
C71—C56—C57—C58	175.7 (2)	C141—C136—C137—C138	0.5 (4)
C56—C57—C58—C59	1.0 (3)	C136—C137—C138—C139	−1.0 (4)
C56—C57—C58—C63	−177.4 (2)	C137—C138—C139—C140	0.7 (4)
C55—C54—C59—C58	−2.6 (4)	C138—C139—C140—C141	0.2 (4)
N8—C54—C59—C58	175.6 (2)	N17—N18—C141—C136	0.3 (3)
C57—C58—C59—C54	1.0 (3)	N17—N18—C141—C140	177.2 (3)
C63—C58—C59—C54	179.4 (2)	N16—C136—C141—N18	0.1 (3)
C55—O7—C60—C61	126.7 (2)	C137—C136—C141—N18	177.7 (2)
C62—O8—C61—O9	2.9 (4)	N16—C136—C141—C140	−177.2 (2)
C62—O8—C61—C60	−175.3 (2)	C137—C136—C141—C140	0.4 (4)
O7—C60—C61—O9	6.1 (4)	C139—C140—C141—N18	−177.4 (3)
O7—C60—C61—O8	−175.72 (19)	C139—C140—C141—C136	−0.8 (4)
C57—C58—C63—C65	−147.5 (2)		
